# Selective photocatalytic CO_2_ reduction in aerobic environment by microporous Pd-porphyrin-based polymers coated hollow TiO_2_

**DOI:** 10.1038/s41467-022-29102-0

**Published:** 2022-03-17

**Authors:** Yajuan Ma, Xiaoxuan Yi, Shaolei Wang, Tao Li, Bien Tan, Chuncheng Chen, Tetsuro Majima, Eric R. Waclawik, Huaiyong Zhu, Jingyu Wang

**Affiliations:** 1 Key Laboratory of Material Chemistry for Energy Conversion and Storage (Ministry of Education), School of Chemistry and Chemical Engineering, Huazhong University of Science and Technology, Wuhan, 430074 China; 2 Key Laboratory of Polyoxometalate Science of Education Institution, Faculty of Chemistry, Northeast Normal University, Changchun, 130024 China; 3 Key Laboratory of Photochemistry, CAS Research/Education Center for Excellence in Molecular Sciences, Institute of Chemistry, Chinese Academy of Sciences, Beijing, 100190 China; 4 School of Chemistry, Physics and Mechanical Engineering, Queensland University of Technology, Brisbane, QLD 4001 Australia

**Keywords:** Photocatalysis, Nanoscale materials, Energy

## Abstract

Direct photocatalytic CO_2_ reduction from primary sources, such as flue gas and air, into fuels, is highly desired, but the thermodynamically favored O_2_ reduction almost completely impedes this process. Herein, we report on the efficacy of a composite photocatalyst prepared by hyper-crosslinking porphyrin-based polymers on hollow TiO_2_ surface and subsequent coordinating with Pd(II). Such composite exhibits high resistance against O_2_ inhibition, leading to 12% conversion yield of CO_2_ from air after 2-h UV-visible light irradiation. In contrast, the CO_2_ reduction over Pd/TiO_2_ without the polymer is severely inhibited by the presence of O_2_ ( ≥ 0.2 %). This study presents a feasible strategy, building Pd(II) sites into CO_2_-adsorptive polymers on hollow TiO_2_ surface, for realizing CO_2_ reduction with H_2_O in an aerobic environment by the high CO_2_/O_2_ adsorption selectivity of polymers and efficient charge separation for CO_2_ reduction and H_2_O oxidation on Pd(II) sites and hollow TiO_2_, respectively.

## Abstract (web summary)

While selective CO_2_ reduction is crucial for its removal from the environment, the presence of O_2_ hinders this process. Here authors show CO_2_ photoreduction in the presence of O_2_ by incorporating Pd(II) sites into a coordination polymer on TiO_2_ to selectively adsorb CO_2_.

## Introduction

Photocatalytic CO_2_ reduction into useful fuels is a promising approach to tackle the challenges of carbon emission and global warming by directly utilizing sustainable solar energy^1–3^. Despite extensive efforts and many attempts at harnessing various semiconductor photocatalysts for CO_2_ reduction, most of the photocatalytic reactions occur only at high CO_2_ concentration and sometimes CO_2_-philic organic solvents are required to make them operate efficiently, due to low CO_2_ uptake of the photocatalysts^1–6^. Physisorptive microporous solids such as microporous organic polymers and metal-organic frameworks have recently emerged as promising candidates to replace aqueous amines for CO_2_ capture and storage^7–11^. Several reports demonstrated the integration of metals into CO_2_-adsorptive materials could convert diluted CO_2_ due to the high CO_2_ uptake and high reduction activity of metals during the photocatalytic reactions^12–14^. However, metal sites suffer from poor H_2_O oxidation activity and highly active for H_2_ evolution from H_2_O, so that these photocatalysts require addition of Ru-containing photosensitizer together with organic sacrificial reagent and solvent^12–14^, which present unsustainable and negative environmental impact issues. More importantly, anaerobic environment is essential to avoid the competitive reaction of oxygen reduction because it is thermodynamically favored compared to CO_2_ reduction^15–20^.

An ideal catalyst is capable of taking gaseous feedstocks^21^. In practice, the CO_2_ concentration in air is as low as 300~400 ppm, and flue gas after fossil fuel combustion typically consists of about 72–77 vol% N_2_, 12–14 vol% CO_2_, 8–10 vol% H_2_O, 3–5 vol% O_2_, and other minor components^21–24^. In air and flue gas, the CO_2_ adsorption and activation on the surface of photocatalysts are low, due to the competitive O_2_ adsorption and reduction, as well as the low CO_2_ concentration^15–20^. Catalytic CO_2_ reduction is strongly influenced by the presence of 5 ppm of O_2_ and completely inhibited in 5 vol% O_2_, because O_2_ reduction is thermodynamically favored compared to CO_2_ reduction^20^. Therefore, to control CO_2_ emission from exhaust gas and reduce CO_2_ concentration in air, developing efficient photocatalysts with selective CO_2_ adsorption and conversion in an aerobic environment remains a challenge.

To address the challenge, we envisioned that significantly increasing CO_2_ concentration around the catalytic active sites for CO_2_ reduction via preferential adsorption of CO_2_ over O_2_ could lessen the inhibitive impact of O_2_ and promoting H_2_O oxidation could increase CO_2_ conversion. High CO_2_ adsorption capability and selectivity of microporous polymers with heterocyclic skeleton and large π-conjugated structure can bring opportunities for directly using low concentration of CO_2_ without separation from aerobic mixtures if the catalytic active sites for CO_2_ reduction are built in the polymer. For the photocatalytic CO_2_ reduction with H_2_O in such an aerobic environment, another essential requirement is to assemble the photocatalytic sites for CO_2_ reduction and H_2_O oxidation for efficient separation of photogenerated electrons and holes, respectively. Meanwhile, H_2_O provides protons for reacting with the intermediates from CO_2_ reduction, increasing CH_4_ production. Electron transfer at the heterointerface between two components is required for the occurrence of CO_2_ reduction and H_2_O oxidation at different active sites in a composite structure.

In this work, a proof-of-concept study was conducted to verify this hypothesis. We prepared a porous composite photocatalyst by in situ hyper-crosslinking porphyrin-based polymers (HPP) on a hollow TiO_2_ surface, followed by loading Pd(II) via coordination with HPP to form the CO_2_ reduction sites (Pd-HPP-TiO_2_). Hollow TiO_2_ was used to increase the heterointerface between TiO_2_ and Pd-HPP. The choice of Pd allows us to confirm the influence of CO_2_ adsorption and charge separation on the reduction. The heteroatom-rich microporous structure can not only improve the capability and selectivity of CO_2_ adsorption in an aerobic environment but also stabilize Pd(II) sites, while anatase TiO_2_ surface is highly efficient for H_2_O oxidation with holes that generated in the valence band from the bandgap excitation. Pd-HPP-TiO_2_ achieves the efficient conversion of CO_2_ in an aerobic environment, i.e., 12 % of CO_2_ in air is converted after 2-h UV-visible light irradiation with a CH_4_ production of 24.3 μmol g^−1^, which is 4.5 times higher than that over Pd/TiO_2_. Based on the catalytic activity, we identify the active sites for photocatalytic CO_2_ reduction and discuss the overall reaction mechanism.

## Results and discussion

### Preparation of porous Pd-HPP-TiO_2_

**Fig. 1:**
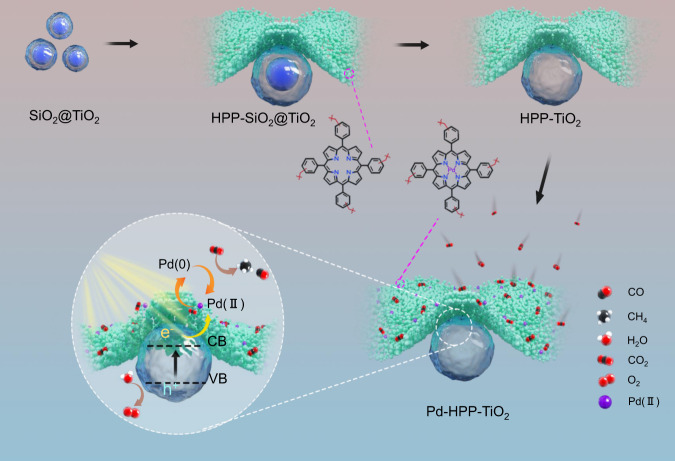
Schematic illustrations. Synthesis of porous Pd-HPP-TiO_2_ and the possible mechanism of photocatalytic CO_2_ reduction. The chemical structures of HPP and Pd-HPP units are provided.

The synthetic processes of porous Pd-HPP-TiO_2_ are depicted in Fig. 1. HPP were knitted together from 5,10,15,20-tetraphenylporphyrin (TPP) building blocks on the surface of core-shell SiO_2_@TiO_2_ with the diameter of 100–150 nm used as solid templates. The SiO_2_ cores were then etched by NaOH solution to produce hollow TiO_2_ with the thickness of about 10 nm coated by layers of HPP with the thickness of about 5–7 nm (HPP-TiO_2_), finally Pd(II) coordinates with the core of porphyrin unit, leading to the formation of porous Pd-HPP-TiO_2_ (details are provided in the Methods). The photocatalytic activity was evaluated in a gas-solid reaction without the addition of photosensitizer or organic sacrificial reagent under UV-visible light irradiation. CH_4_ and CO were detected as the main products, in accordance with the results from many gas-solid reactions^25,26^. To clarify the effect of HPP, Pd/TiO_2_ was synthesized as a control by photo-deposition of Pd nanoparticles on the surface of hollow TiO_2_.

### Photocatalytic CO_2_ reduction

**Fig. 2:**
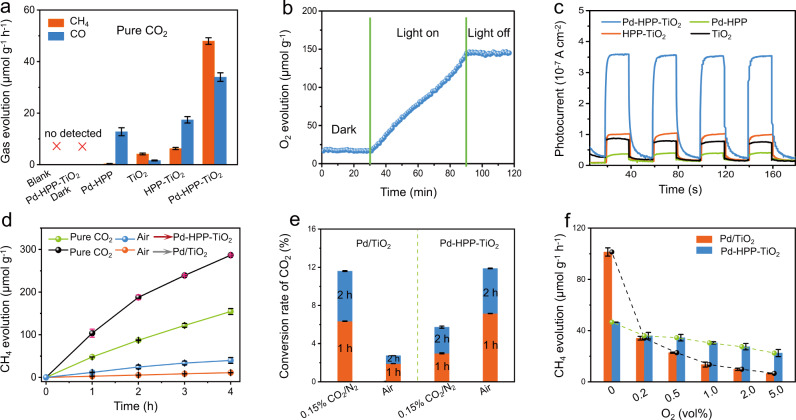
Evaluation of photocatalytic CO_2_ reduction. **a** The evolution rates of CH_4_ and CO in pure CO_2_. **b** On-line monitoring of O_2_ evolution during the photocatalytic reaction over Pd-HPP-TiO_2_. **c** Photocurrent response during light on-off cycling. **d** Comparison of the CH_4_ evolution rates in pure CO_2_ and air. **e** The conversion yield of CO_2_ by measuring the CO_2_ concentration. **f** Effect of O_2_ concentration (vol%) in CO_2_/O_2_ gas mixture on the CH_4_ evolution rate. The results in (**a**, **d**, **e**, **f**) are the average values of three parallel experiments. The error bar represents the standard deviation of the measurements.

Figure 2a shows the comparison of CH_4_ and CO evolution rates over a series of photocatalysts in pure CO_2_. Hollow TiO_2_ presented evolution rates of 4.2 and 1.6 μmol g^−1^ h^−1^ for CH_4_ and CO, respectively. HPP-TiO_2_ caused a moderate increase of CO evolution rate, mainly arising from the introduction of an abundance of micropores on HPP, favoring the CO_2_ uptake. When building Pd(II) sites into HPP-TiO_2_, the CO_2_ reduction efficiency was further enhanced, reaching high evolution rates of 48.0 and 34.0 μmol g^−1^ h^−1^ (average value within 4 h) for CH_4_ and CO, respectively. The comparison to the reported results under similar reaction conditions suggests the excellent photocatalytic activity of porous Pd-HPP-TiO_2_ composite (Supplementary Table 1). The high selectivity as 59% for CH_4_ production over Pd-HPP-TiO_2_ can be attributed to the Pd(II) sites with sufficient energy overcoming the Schottky barrier with TiO_2_ and improving the charge separation efficiency^27^. In a long-term test, Pd-HPP-TiO_2_ showed continuous CH_4_ and CO production up to 20 h under UV-visible light irradiation (Supplementary Fig. 1). Although there is somewhat loss in catalytic activity, the superior performance of porous Pd-HPP-TiO_2_ to Pd/TiO_2_ during long-term photocatalytic reaction suggests that the introduction of microporous HPP greatly contributes to stabilizing Pd(II) sites. No detectable H_2_ during the photocatalytic reaction suggests the higher CO_2_ reduction selectivity than H_2_O reduction. It is noted that Pd-HPP exhibits the ability to catalyze the conversion of CO_2_ to CO, which is consistent with the reports on various metal complexes^12–14,28^. Only CH_4_ was produced over the photocatalysts containing TiO_2_. The surface of anatase TiO_2_ efficiently adsorbs H_2_O to facilitate H_2_O oxidation and provide protons for the CO_2_ reduction to yield CH_4_^29–31^. The efficient consumption of light-generated holes on the TiO_2_ surface can accelerate the overall reaction.

The photocatalytic CO_2_ reduction was confirmed by a series of control experiments, (1) dark reaction, (2) without photocatalyst, (3) in N_2_, and (4) isotopic label using ^13^CO_2_. No detectable product in the dark or the absence of photocatalysts indicates that the CO_2_ reduction proceeded as a light driven catalytic process (Fig. 2a). Upon replacing CO_2_ with N_2_, trace amounts of CH_4_ and CO were detected after the photocatalytic reaction (Supplementary Fig. 2), presumably due to the slight decomposition of HPP and the presence of pre-adsorbed CO_2_ on the photocatalyst or reactor surface. Isotopically labeled ^13^CO_2_ (^13^C enrichment of ≥ 97%) was used as the reactant to study the origin of products. According to the ion fragment analysis, the peaks at 2.5 min and 7.2 min in the gas chromatography could be assigned to CH_4_ and CO, respectively. As compared to the signals of products under ^12^CO_2_, the appearance of ion fragment peaks at m/z = 17 and 29 reveals that the produced ^13^CH_4_ and ^13^CO originated from ^13^CO_2_ reduction over Pd-HPP-TiO_2_ (Supplementary Fig. 3). The overall reaction involves CO_2_ reduction and H_2_O oxidation cycles to produce CH_4_, CO, and O_2_, respectively, but their concentration changes in the whole cycle have seldom been measured in the literature^32^. The in-situ monitoring of O_2_ evolution during the photocatalytic reaction was performed to further verify the CO_2_ reduction by H_2_O (Fig. 2b). When the experiment was conducted in the dark, low O_2_ concentration in the reaction remained unchanged and came from the residual air, which was not completely removed by the degassing procedure. By way of contrast, during the photocatalytic reaction, the concentration of O_2_ increased linearly with an evolution rate of 127 μmol g^−1^ h^−1^. Thus, the electrons (e^−^) being provided from the 4-e^−^ oxidation are comparable to the total electrons for CH_4_ and CO production via 8-e^−^ and 2-e^−^ reduction processes. When UV-visible light irradiation was turned off, the O_2_ concentration remained constant. This result illustrates that CO_2_ is reduced by H_2_O during photocatalytic reaction over Pd-HPP-TiO_2_ and that the backward reaction does not take place.

As for the photocatalytic CO_2_ reduction, the reaction involved several steps, light absorption to generate electrons and holes in TiO_2_, electron transfer from the conduction band of TiO_2_ to Pd-HPP, electron trapping at the catalytic Pd(II) sites in Pd-HPP, reduction of the adsorbed CO_2_ on Pd(II) sites^33^. The charge separation and charge transfer efficiency were investigated by electrochemical, photochemical, and photoelectrochemical measurements. The hollow TiO_2_ displays a large semicircle arc at the high frequency of electrochemical impedance spectrum (EIS), indicating less electronic conductivity and larger electron transfer resistance (*R*_ct_) (Supplementary Fig. 4). The value of *R*_ct_ in the EIS of HPP-TiO_2_ is smaller than those of both TiO_2_ and Pd-HPP, indicating that the interface between the polymer and TiO_2_ facilitates the electron transfer. The smallest value of *R*_ct_ in the EIS of Pd-HPP-TiO_2_ illustrates the efficient interfacial electron transfer by the surface binding HPP coordinating with Pd on hollow TiO_2_. When coating with Pd-HPP, the photoluminescence of TiO_2_ was almost quenched, indicating the efficient suppression of photogenerated charge recombination through radiative pathways (Supplementary Fig. 5). The photogenerated electrons are expected to transfer from the photoexcited TiO_2_ to Pd(II) sites in HPP, leading to effective separation of electrons from the holes left in TiO_2_. In addition, the introduction of Pd enhanced the interaction with gas molecules such as O_2_ and CO_2_ from air, which also causes the quenching of photoluminescence on TiO_2_ surface^34–36^. Similar results for Pd/TiO_2_ to Pd-HPP-TiO_2_ in Supplementary Figs. 4 and 5 reveal that Pd in Pd-HPP contributes to the efficient charge separation. The amperometric signals provide further information on the relative efficiency of the electron transfer in the materials under UV-visible light irradiation. The electronic conductivity of Pd-HPP appears to be higher than TiO_2_, and Pd-HPP exhibits the lowest photocurrent among the samples (Fig. 2c). Generally, the photoinduced charge separation in organic polymers does not occur dominantly as compared with exciton migration, leading to the lower capability as redox photocatalysts^37^. Although Pd-HPP possesses strong absorption in the visible region (Supplementary Fig. 6), both Pd-HPP and Pd-HPP-TiO_2_ exhibit low photocatalytic activity for CO_2_ reduction under visible light irradiation, suggesting the low efficiency of charge separation in HPP (Supplementary Fig. 7). It is found that such results are comparable to the recently reported analogous polymer photocatalyst^38^. The comparison of visible light driven CO_2_ reduction to that under UV-visible light is presented in Supplementary Fig. 8. Thus, the photocatalytic CO_2_ reduction reaction over Pd-HPP-TiO_2_ depended on UV light of UV-visible light irradiation. The visible light is absorbed by HPP, and most of photons absorbed are changed to heat. When TiO_2_ is irradiated with UV light to generate electrons and holes in conduction band and valence band, respectively, the electrons transfer at the heterointerface to HPP with π-conjugated structure and can be trapped at Pd(II). It is well know that metals work as electron trap sites to enhance the charge separation efficiency^39^. The highest photocurrent of Pd-HPP-TiO_2_ can be attributed to the photogenerated electrons transferring from TiO_2_ to Pd-HPP and to be trapped at Pd. The order of gas evolution rates shown in Fig. 2a is consistent with that of the photocurrent in Fig. 2c, suggesting that efficient electron transfer in Pd-HPP is the dominant influence on the photocatalytic activity with pure CO_2_.

Pd in Pd-HPP increases the charge separation efficiency, while Pd does not response to the selective CO_2_ reduction in an aerobic environment. In pure CO_2_, Pd/TiO_2_ exhibits high activity for CH_4_ production with a rate of 104 μmol g^−1^ h^−1^ compared to Pd-HPP-TiO_2_ (48.0 μmol g^−1^ h^−1^), as shown in Fig. 2d. In the case of similar Pd loading, the higher rate over Pd/TiO_2_ is ascribed to Pd-HPP absorbing light in a part (Supplementary Fig. 6) and thus decreasing absorbed photon numbers by TiO_2_. Besides, the light-induced electron transfer from TiO_2_ to Pd in Pd/TiO_2_ is more efficient compared with that in Pd-HPP-TiO_2_, in which Pd sites do not directly contact with hollow TiO_2_. When the reaction proceeded in diluted CO_2_ (diluted in N_2_), the CH_4_ evolution rate over Pd/TiO_2_ was higher than that over Pd-HPP-TiO_2_ (Supplementary Fig. 9a), but the difference between them was less with decreasing the CO_2_ concentration (Supplementary Fig. 9b), due to the enrichment of low CO_2_ concentration by the abundant micropores of HPP. The activities for two photocatalysts were close each other using the synthetic gas containing 0.03 vol% CO_2_ (approximate concentration of air, Supplementary Fig. 9b). However, in air, the CO_2_ reduction was almost completely inhibited over Pd/TiO_2_, while it still proceeded over Pd-HPP-TiO_2_ with the evolution rates of 12.2 μmol g^−1^ h^−1^ and 4.9 μmol g^−1^ h^−1^ for CH_4_ and CO production, respectively (Fig. 2d and Supplementary Table 2). The calculated conversion yields of CO_2_ over two catalysts in air and a mixture of CO_2_ and N_2_ are compared in Fig. 2e. Monitoring of change in CO_2_ concentration is important in providing direct evidence for the CO_2_ conversion, but it has been seldom achieved in the literatures because the change is negligibly little in pure CO_2_. It is noted that the reduction efficiency in the gas mixture of 0.15 vol% CO_2_ in N_2_ is close to that in pure CO_2_, and the change in CO_2_ concentration is large enough to calculate the conversion yield, as listed in Supplementary Table 3. Pd/TiO_2_ is more efficient than Pd-HPP-TiO_2_ in an anaerobic environment, while the reverse results were observed in air; CO_2_ conversion yields of 12% and 2.7% over Pd-HPP-TiO_2_ and Pd/TiO_2_, respectively, after 2 h UV-visible light irradiation. The yield of 12% is the highest among the CO_2_ conversions in air reported in the literatures (Supplementary Table 2). The difference between the CO_2_ conversion yields in 0.15 vol% CO_2_ in N_2_ and air is resulted from the absence and presence of O_2_, respectively. Thus, we investigated the effect of O_2_ concentration on the photocatalytic reaction. As can be seen in Fig. 2f, for Pd/TiO_2_, the presence of 0.2 vol% O_2_ suppressed the CH_4_ evolution rate, and the presence of 5 vol% O_2_ dropped it steeply to 6% of that in pure CO_2_^5^. Interestingly, the negative effect of O_2_ on the CH_4_ evolution is significantly less over Pd-HPP-TiO_2_: the presence of 5 vol% O_2_ decreased it to 46% of that in pure CO_2_.

### Porosity and gas uptake

**Fig. 3:**
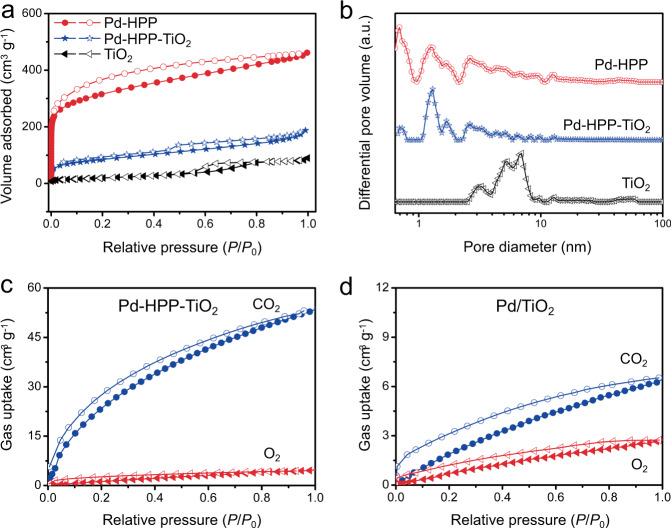
Surface porosity and gas uptake. (**a**) N_2_ adsorption-desorption isotherms and (**b**) pore size distribution plots of hollow TiO_2_, Pd-HPP, and Pd-HPP-TiO_2_. Comparisons in CO_2_ and O_2_ uptake of (**c**) Pd-HPP-TiO_2_ and (**d**) Pd/TiO_2_ at 273 K.

For heterogeneous catalysis, the reaction rate is usually proportional to the surface coverage of reactants on the catalyst, so the CO_2_ conversion efficiency particularly relies on the CO_2_ adsorption on the photocatalysts^33,40,41^. The surface properties including porosity as well as the CO_2_ adsorption capability and selectivity of as-prepared samples were investigated. As shown in Fig. 3a, the N_2_ adsorption-desorption isotherms of Pd-HPP and Pd-HPP-TiO_2_ exhibit a steep increase at relative low pressure (*P*/*P*_0_ < 0.001) and an obvious hysteresis at medium pressure, which indicate the existence of abundant micropores and mesopores^42^. This result may be due to a fast rate of hyper-crosslinking and the low degree of free packing for building blocks by Friedel-Crafts alkylation reaction. In contrast, pure hollow TiO_2_ shows the character of type IV isotherm with a hysteresis loop at medium pressure, which suggests the formation of mesoporous structure and gives a Brunauer–Emmett–Teller surface area (*S*_BET_) of 75 m^2^ g^−1^. Owing to the high microporosity of HPP (0.7 and 1.3 nm), Pd-HPP-TiO_2_ has a large surface area of 323 m^2^ g^−1^ and micropore volume of 0.22 cm^3^ g^−1^ (Supplementary Table 4). The introduction of TiO_2_ caused a moderate decrease of the ultra-micropore of HPP, while micropores centered at 1.3 nm were largely remained (Fig. 3b). The microporous nature of Pd-HPP-TiO_2_ causes the CO_2_ enrichment around the catalytic active Pd sites in Pd-HPP. The CO_2_ adsorption capability of Pd-HPP-TiO_2_ reaches as high as 54.0 cm^3^ g^−1^ at 1.0 bar and 273 K, which is 4.9 times higher than that of TiO_2_ (Fig. 3c and Supplementary Fig. 10). In contrast, the as-obtained Pd/TiO_2_ shows a low CO_2_ uptake of 6.5 cm^3^ g^−1^ under similar conditions. To study the effect of porphyrin concentration on the adsorption of CO_2_ and photocatalytic reaction, we have prepared porous Pd-HPP-TiO_2_ composites with different mass percentage of porphyrin unit by adjusting the adding amount of porphyrin monomer. The CO_2_ uptake of porous Pd-HPP-TiO_2_ composites with 53.8 and 74.9 wt% of Pd-HPP was presented in Supplementary Fig. 11. According to the adding amount of porphyrin monomer and the yield of resulted polymer, the mass percentage of porphyrin unit in Pd-HPP is calculated to be about 70.5%. Thus the molar ratios of porphyrin unit/adsorbed CO_2_ can be calculated and compared in Supplementary Table 5. The results suggest that the ratio of porphyrin/CO_2_ almost keeps constant. A little lower porphyrin/CO_2_ ratio in Pd-HPP-TiO_2_ composites than that in pure Pd-HPP is presumably due to the introduction of TiO_2_ slightly blocking the crosslinking of porphyrin monomer. It can be concluded that the adsorption of CO_2_ molecules strongly depends on the porphyrin content. Besides the CO_2_ adsorption, electron generation on TiO_2_ photocatalyst and trapping by Pd(II) sites are crucial processes that involved in the photocatalytic reactions. The result of photocatalytic CO_2_ reduction in Supplementary Fig. 12 reveals that there is an appropriate porphyrin content that balanced the CO_2_ adsorption and conversion efficiency.

The selectivity ratio of CO_2_/O_2_ over Pd/TiO_2_ is 3.1 calculated by initial slopes of adsorption isotherms in the low pressure region (Fig. 3d and Supplementary Fig. 13), indicating a mediocre CO_2_ adsorption selectivity in the presence of O_2_. Interestingly, Pd-HPP-TiO_2_ exhibits a high CO_2_/O_2_ selectivity ratio of 23.9. Moreover, CO_2_ has delocalized π-bonds with higher quadrupole moment (−13.4 × 10^−40^ C m^2^) than O_2_ ( − 1.03 × 10^−40^ C m^2^)^43,44^. Introducing porphyrin with a core of four pyrrole rings as the building blocks into microporous materials endows them polarizing N-containing groups and large π-conjugated structure, which could response to the enhanced interaction with CO_2_^9–11,45^. The high affinity for CO_2_ instead of O_2_ for microporous Pd-HPP-TiO_2_ is consistent with the selective CO_2_ adsorption and reduction. Thus the photocatalytic CO_2_ reduction is achieved in an aerobic environment by taking the advantage of selective CO_2_ adsorption in microporous HPP.

### Structural analysis

**Fig. 4:**
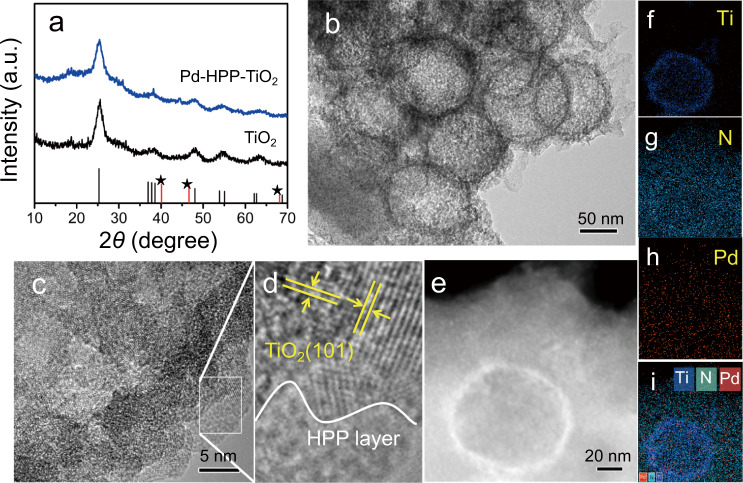
Crystal and morphology characterizations. **a** XRD patterns of TiO_2_ and Pd-HPP-TiO_2_. The vertical lines are the position and intensity of anatase TiO_2_ (JCPDS 21-1272) and Pd (labeled by the star, JCPDS 46-1043). **b** TEM, **c**, **d** HRTEM, **e** STEM, and **f**–**i** the corresponding EDX element mapping images of Pd-HPP-TiO_2_.

Structural characterizations provide more information for understanding the selective CO_2_ adsorption and conversion. Pd-HPP-TiO_2_ displays the X-ray diffraction (XRD) peaks of pure TiO_2_ anatase (Fig. 4a). No diffraction peak of Pd crystal indicates Pd (II) coordinates to the porphyrin in Pd-HPP. The observation by scanning electron microscopy (SEM) reveals the morphology of the core-shell SiO_2_@TiO_2_ to have a uniform size of 100~200 nm for template-assisted knitting of TPP (Supplementary Fig. 14). The transmission electron microscopy (TEM) and high-resolution TEM (HRTEM) images indicate that hollow TiO_2_ has the characteristic lattice plane of anatase TiO_2_ (101), coated by Pd-HPP (Fig. 4b–d and Supplementary Fig. 15). The structure of Pd-HPP-TiO_2_ was observed by TEM, and no Pd nanoparticle was detected in the HRTEM image, which is consistent with the XRD analysis. As further evidence, the elemental distributions were analyzed by scanning transmission electron microscopy (STEM) and energy-dispersive X-ray (EDX) mapping tests. Figure 4e–i display that hollow TiO_2_ is embedded in Pd-HPP with a homogeneous distribution of C, N, and Pd elements.

**Fig. 5:**
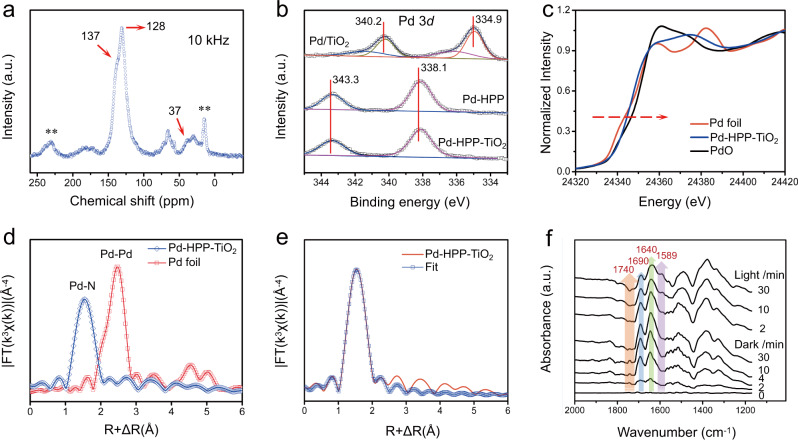
Chemical structure analysis and surface species evolution of Pd-HPP-TiO_2_. **a** Solid-state ^13^C CP-MAS NMR spectrum. **b** Comparison of Pd 3*d* XPS spectra for three photocatalysts. **c** Pd K-edge XANES and **d** Fourier transformed EXAFS spectra of Pd-HPP-TiO_2_ and references. **e** Fourier transformed EXAFS spectrum of Pd-HPP-TiO_2_ and fitting curve. **f** In situ DRIFTS test of gas adsorption on Pd-HPP-TiO_2_ in the dark and during the photocatalytic CO_2_ reduction under UV-visible light irradiation.

The chemical structure of Pd-HPP-TiO_2_ was investigated by Fourier transform infrared (FT-IR) absorption, solid-state ^13^C cross-polarization magic-angle spinning nuclear magnetic resonance (CP-MAS NMR), and X-ray photoelectron spectroscopy (XPS) measurements. As shown in FT-IR spectrum, the C-H stretching band at 2920–2960 cm^−1^ indicates the methylene linkage of HPP by solvent knitting (Supplementary Fig. 16)^46^. The broad bands of Ti-O-Ti stretching vibrations at 400–1000 cm^−1^ are observed for TiO_2_ and Pd-HPP-TiO_2_. The CP-MAS NMR spectrum of Pd-HPP-TiO_2_ indicates evidence for the hyper-crosslinking process at the molecular level. The resonance peaks at 128, 137, and 146 ppm are attributed to the carbon atoms in the benzene ring and porphyrin ring (Fig. 5a). The peak with a chemical shift at 37 ppm corresponds to the methylene linkers, indicating the successful linking of TPP via the Friedel-Crafts reaction^47^. The chemical states of elements were analyzed by the XPS spectrum to display the presence of C, N, Ti, O, and Pd in the corresponding samples and the coexistence of them in Pd-HPP-TiO_2_ (Supplementary Fig. 17). The high-resolution Pd 3*d* spectra in Fig. 5b show distinct doublet peaks at 343.3 and 338.1 eV, assigned to 3*d*_5/2_ and 3*d*_3/2_ of the coordinated Pd(II)^48,49^. In contrast, metallic Pd is predominantly observed in Pd/TiO_2_ XPS, together with a weak shoulder peak of the adsorbed Pd^2+^ that remained without reducing^49,50^. Combined with the Pd-N signal in N 1 *s* spectrum of Pd-HPP-TiO_2_ (Supplementary Fig. 17), it is deduced that Pd coordinates successfully with the core of the porphyrin unit as Pd(II) but not as free Pd^2+^ or metallic Pd. The formation of Pd-HPP on hollow TiO_2_ caused the binding energy of Ti 2*p* shifting to 0.7-eV higher energy (Supplementary Fig. 17). This shift reflects that the electron density of Ti is decreased by the electronic interaction with Pd-HPP, which is favorable for the electron transfer from TiO_2_ to Pd-HPP during the photocatalytic reactions under UV-visible light irradiation.

Synchrotron-based X-ray absorption spectroscopy was employed to provide further information on the valence state. Figure 5c shows the Pd K-edge X-ray absorption near-edge structure (XANES) spectra. The absorption edge energy of Pd-HPP-TiO_2_ is close to that of PdO but higher than that of Pd foil, confirming Pd(II) in Pd-HPP-TiO_2_. Fourier transform of the extended X-ray absorption fine structure (EXAFS) displays the main peak at 1.5 Å for Pd-HPP-TiO_2_ (Fig. 5d), arising from Pd-N bonding. No obvious peak was observed at the Pd-Pd position (2.5 Å) of Pd foil, indicating that Pd(II) sites were dispersed in Pd-HPP-TiO_2_^48,51^. The structural parameters were obtained by the quantitative EXAFS curve fitting (Fig. 5e). Supplementary Table 6 reveals that the coordination number of Pd in Pd-HPP-TiO_2_ is close to 4.0, indicating that Pd(II) coordinates to four N atoms of the porphyrin. The measured Pd-N bond distance of 2.03 Å is also close to the reported results of Pd-N_4_ center^48,49^. Inductively coupled plasma mass spectrometry (ICP-MS) provides the accurate element composition, showing that the weight ratios of TiO_2_ and Pd were 34.4% and 2.72%, respectively (Supplementary Table 7). Meanwhile, the similar Pd content was ensured in the control (Pd/TiO_2_) to avoid the effect of metal amount on the photocatalytic activity. Based on the above characterizations, it is concluded that HPP is successfully formed on the surface of hollow TiO_2_ and then Pd(II) coordinates to the porphyrin core of HPP to form Pd-HPP-TiO_2_.

### Photocatalytic mechanism of CO_2_ reduction with H_2_O

To clarify the reaction pathway, the reaction intermediates of CO_2_ adsorption and photocatalytic reduction on the surface of Pd-HPP-TiO_2_ were monitored by in-situ diffuse reflectance infrared Fourier transform spectra (DRIFTS). The spectra demonstrate the adsorption of CO_2_ and H_2_O on Pd-HPP-TiO_2_ in the dark. The absorption band in the range of 3500–3800 cm^−1^ are in good agreement with those assigned to the stretching vibrations of surface-bonded OH groups and H_2_O, suggesting the H_2_O adsorption on the catalyst surface (Supplementary Fig. 18)^52,53^. The peaks at 1740, 1690, and 1640 cm^−1^ can be assigned to the surface adsorbed carbonate species (Fig. 5f)^54,55^_._ Under UV-visible light irradiation, the peaks at 1690 and 1640 cm^−1^ were significantly weakened. Meanwhile, the peak at 1740 cm^−1^ first became flat at 2 min and then changed to a negative peak with prolonged irradiation, which could be explained by the existence of pre-adsorbed carbonate species on the Pd-HPP-TiO_2_ surface before collecting the baseline due to its high CO_2_ uptake. The results indicate the efficient consumption of surface carbonate during the photocatalytic reaction. Meanwhile, a new peak at 1589 cm^−1^ emerged in the spectra is suggested to be the C = O stretching vibration of *COOH groups, which was the vital intermediate for *CO formation and then transformed to CO and other fuels.^56–58^ According to the detailed studies on the mechanism of CO_2_ reduction, there are two possible pathways, i.e., one is the formaldehyde pathway and the other is the carbene pathway^59–61^. Although formaldehyde and methanol have been reported as products in some setups, they are not detected in this work. The photocatalytic CO_2_ reduction in the gas-solid reaction can normally form CO and CH_4_.^25,26^ Therefore, the CO_2_ reduction is more likely to be a carbene pathway as CO_2_ → COOH → CO → •C → •CH_3_ → CH_4_. The CO_2_ molecules are activated at Pd(II) sites in Pd-HPP and then react with the proton and electron to produce the intermediate *COOH. Subsequent reaction with the electron and proton results in the splitting of *COOH into *CO and H_2_O. At this time, part of CO is produced by *CO desorption. Since Pd can also function efficiently for the decomposition of H_2_O to generate Pd-H species^62,63^, the adsorbed *CO on Pd will be further react with the dissociated H to the formation of •C radicals, which can successively combine with •H radicals, thereby forming •CH, •CH_2_, •CH_3_, and finally CH_4_ product^53,64–66^.

The cycling experiment and related characterizations were carried out to investigate the photocatalytic sites of CH_4_ evolution and the stability of Pd-HPP-TiO_2_. As shown in Supplementary Fig. 19, Pd-HPP-TiO_2_ showed about 80% of the initial photocatalytic activity for the CO_2_ conversion after five consecutive cycles. The thermal stability of HPP is maintained up to 330 °C, as confirmed by thermogravimetric analysis (Supplementary Fig. 20). The comparison in FT-IR spectra of Pd-HPP-TiO_2_ before and after the photocatalytic reaction reveals no obvious change in the chemical structure of Pd-HPP (Supplementary Fig. 21). A new peak at 40.1° is observed in the XRD pattern of Pd-HPP-TiO_2_ after the cycling test to be assigned to Pd(0) (Supplementary Fig. 22), implying that a fraction of the coordinated Pd(II) was reduced to Pd(0) dissociating from the coordination sites to form Pd nanoparticles. XPS analysis in Supplementary Fig. 23a corroborates the reduction, leading to a decrease of the photocatalytic activity during the recycle. Such reduction suggests the electron trapped at Pd(II) for CO_2_ reduction^67^. After five cycles, ~79% Pd existed as Pd(II) owing to the coordination to the porphyrin in microporous HPP, indicating the considerable stability of Pd-HPP-TiO_2_ structure during the photocatalytic reaction. Pd nanoparticles are less active for the photocatalytic CO_2_ reduction in air (Fig. 2e), compared to Pd(II) in Pd-HPP-TiO_2_. Therefore, a tentative mechanism of photocatalytic CO_2_ reduction over Pd-HPP-TiO_2_ is proposed in Fig. 1. Benefiting from the high CO_2_ adsorption capability and selectivity of HPP, CO_2_ can be selectively enriched in Pd-HPP. Under UV light irradiation, the photogenerated electrons in the conduction band of TiO_2_ transfer efficiently to Pd-HPP, the electrons are trapped at the coordinated Pd(II) in Pd-HPP, and the adsorbed CO_2_ on Pd(II) is reduced to produce CH_4_ and CO accompanied by the recovery of Pd(II), while the holes in the valence band of TiO_2_ can oxidize water that adsorbed on hollow TiO_2_ to produce O_2_. The simultaneous monitoring of concentrations of CO_2_ (decreased), CH_4_, CO, and O_2_ (produced), corroborates the overall redox reaction over Pd-HPP-TiO_2_, applicable to aerobic environment, especially for flue gas with 3~5 vol% O_2_ and air with 300~400 ppm of CO_2_ and ~21 vol% O_2_ content.

In summary, we have demonstrated that Pd-HPP-TiO_2_, constructed based on higher CO_2_ adsorption capability than O_2_ and efficient charge separation for CO_2_ reduction and H_2_O oxidation, exhibits high photocatalytic activity for CO_2_ reduction in an aerobic environment. In the presence of 5 vol% O_2_, the CO_2_ reduction over a catalyst without HPP (Pd/TiO_2_) steeply drops to 6% of that in pure CO_2_. In contrast, the O_2_ inhibition is significantly less over Pd-HPP-TiO_2_, which maintained 46% of the CH_4_ evolution rate in pure CO_2_. Pd-HPP-TiO_2_ shows the photocatalytic activity even in air with the CO_2_ conversion yield of 12% and the CH_4_ production of 24.3 μmol g^−1^ after 2 h UV-visible light irradiation, 4.5 times higher than those over Pd/TiO_2_. The HPP layer effectively enriches CO_2_ at Pd(II) to lessen the O_2_ reduction. Water adsorbed on TiO_2_ is oxidized by the holes in the valence band of TiO_2_, leading to reduce the charge recombination and enhance CO_2_ conversion. This study presents an insight into realizing the photocatalytic selective CO_2_ reduction for effectively reducing CO_2_ concentration in air or flue gas and producing valuable solar fuels as well.

## Methods

### Preparation of core-shell SiO_2_@TiO_2_ and hollow TiO_2_ sphere

The preparation of SiO_2_@TiO_2_ was referenced in the literature^68^. In a 100 mL round bottom flask, a mixture containing 79 mL of ethanol, 3.9 mL of ammonia solution, and 1.4 mL of water was mixed with 1.0 g of SiO_2_ nanoparticles with diameter of about 100 nm to obtain a SiO_2_ colloidal solution. Then, 28 mL of acetonitrile was added to the above mixture with stirring at 4 °C. A solution containing 36 mL of ethanol, 12 mL of acetonitrile, and 1 mL of titanium isoporpoxide was added dropwise to the colloidal SiO_2_ solution. The mixture was stirred vigorously for 12 h, and the resulting white solution was dried in an oven at 80 °C. After calcining the solution at 600 °C for 6 h, core-shell SiO_2_@TiO_2_ with diameter of 100–150 nm was obtained as a white powder. Hollow TiO_2_ with thickness of about 10 nm were prepared by etching SiO_2_@TiO_2_ in 10 mL of 2.5 M NaOH solution for 2 days.

### Preparation of Pd-HPP-TiO_2_

HPP-TiO_2_ was synthesized by an in-situ knitting method using SiO_2_@TiO_2_, 5,10,15,20-tetraphenylporphyrin (TPP), and dichloromethane (DCM, 8 mL) as a solid template, building block, and solvent, respectively. After uniform dispersion of SiO_2_@TiO_2_ and TPP in DCM, AlCl_3_ catalyst was added at 0 °C with constant stirring. The reaction mixture was stirred at 0 °C for 4 h, 30 °C for 8 h, 40 °C for 12 h, 60 °C for 12 h, and 80 °C for 24 h under the protection of N_2_ gas. Then, the sample was filtrated and washed twice with water and twice with ethanol, followed by further purification by extracting with ethanol for 2 days. Finally, the obtained solid was dried in a vacuum drying oven at 60 °C for 24 h, and etched in a 2.5 M NaOH solution for 2 days to yield HPP-TiO_2_. 40 mg of HPP-TiO_2_ or HPP was dispersed in 4 mL of acetonitrile and ultra-sounded for 5 min to obtain a homogeneous solution. Then 3 mL of H_2_PdCl_4_ solution (1.08 mg mL^−1^ Pd) was added and kept at 40 °C for 12 h. After the reaction stopped, the product was washed twice with water and twice with acetone, and vacuum dried at 60 °C overnight.

### Preparation of Pd/TiO_2_ as a reference

Pd nanoparticles were deposited on the surface of hollow TiO_2_ from the photoreduction of Pd(II) on TiO_2_: 100 mg of hollow TiO_2_ was dispersed in 90 mL of deionized water and 10 mL of methanol. After ultrasonic treatment for 20 min, a certain amount of H_2_PdCl_4_ solution was added. Then, N_2_ was continuously flowed into the solution for 20 min to ensure N_2_-saturation. A mercury lamp was used for the photoreduction of H_2_PdCl_4_ on hollow TiO_2_. After the photocatalytic reaction for 4 h, the solution was centrifuged and washed three times with ethanol and twice with water, and then dried overnight in a vacuum at 60 °C to yield Pd/TiO_2_ as a gray powder.

### Characterizations

Gas (N_2_, CO_2_, O_2_) adsorption-desorption isotherms were analyzed by TriStar II 3flex adsorption apparatus (Micromeritics, USA). Samples were degassed at 100 °C for 12 h under vacuum before analysis. The structure and crystallinity of the samples were characterized using X-ray diffraction (XRD) analysis on an X-Pert PRO diffractometer with Cu-Kα radiation. The field emission scanning electron microscopy (FESEM) images were recorded by using a field emission scanning electron microscope (FEI Sirion 200, USA) at 10 kV. The high-resolution transmission electron microscopy (HR-TEM) and scanning transmission electron microscopy (STEM) images of samples were recorded on Tecnai G2 F30 microscope (FEI, Holland). The FT-IR experiment was conducted on a Bruker ALPHA Fourier transform infrared spectrometer. The Solid-state ^13^C CP/MAS NMR spectra were tested on a WB 400 MHz Bruker Avance II spectrometer. The ^13^C CP/MAS NMR spectra were collected on a 4 mm double resonance MAS probe at a rotation rate of 10 kHz. X-ray photoelectron spectroscopy (XPS) was measured with a monochromatic Mg Kα source on Thermo VG scientific ESCA MultiLab-2000, and the data were calibrated according to the C (carbon) 1 s peak (binding energy = 284.6 eV). The X-ray absorption spectra were collected on the beamline BL01C1 in NSRRC, and were provided technical support by Ceshigo Research Service “www.ceshigo.com”. The radiation was monochromatized by a Si (111) double-crystal monochromator. XANES and EXAFS data reduction and analysis were processed by Athena software. The actual contents of Ti and different metals were measured by inductively coupled plasma mass spectrometry (NexION 300X, Perkin Elmer, USA). Photoluminescence (PL) emission spectra were collected by a Hitachi F-7000 spectrofluorometer at the excitation wavelength of 360 nm. UV-vis diffuse reflectance spectra (DRS) were obtained using a UV-vis spectrophotometer (UV-3600, Shimadzu, Japan). The reduction products from ^13^CO_2_ were analyzed by HP 5973 gas chromatography-mass spectrometry (GC-MS). Thermogravimetric analysis (TGA) was carried out in N_2_ and air from room temperature to 850 °C using the Perkin Elmer instrument Pyris1 TGA with a heating rate of 10 °C min^−1^. The electrochemical and photoelectrochemical properties of the sample were tested using an electrochemical workstation (CHI650E, Chenhua Com., China) with a standard three-electrode system. A Pt wire and Ag/AgCl were used as the counter and reference electrodes, respectively. 5 mg of a catalyst was dispersed into 1 mL of 1:1 isopropanol/H_2_O containing 10 μL of Nafion. Then, 50 μL of the above suspension was coated on an ITO glass as a working electrode. Electrochemical impedance spectra (EIS) were obtained in 0.1 M KCl electrolyte containing 5 mM Fe(CN)_6_^3−^/Fe(CN)_6_^4−^. Photocurrent signals were detected in 1 M Na_2_SO_4_ solution during light-on and light-off cycles.

### Photocatalytic CO_2_ reduction

30 mg of powder sample was dispersed on the middle of the culture ware, placed in the sealed custom-made glass vessel, 10 cm away from the light source. The photocatalytic CO_2_ reduction reaction was performed under 1 atm of a certain atmosphere (pure CO_2_, CO_2_/O_2_ mixed gas, or air). 2 mL of water, as proton source for the CH_4_ production, was dropped to the bottom of glass vessel and vaporized on standing. The reaction mixture was irradiated using a 300 W Xenon lamp (PLS-SXE300D, Beijing Perfectlight Technology, China) as light source. The full spectrum locates in the UV-visible light region (325~780 nm), as shown in Supplementary Fig. 24. For the visible light driven experiment, a cut-off filter of 420 nm was equipped with the lamp. The generated gases were analyzed by a gas chromatography analyzer (Shimadzu GC-2014C, Japan) with a flame ionization detector (FID) and an optic fiber oxygen sensor (Ocean-Optics, UK). The yield of CO_2_ conversion was conducted in 0.2 vol% CO_2_ in N_2_ or air under irradiation for 4 h. To exclude the self-decomposition of samples, the sample was firstly irradiated for 4 h under N_2_ to confirm the stability.

## Supplementary information

Supplementary Information

Peer Review File

## Data Availability

The data supporting the findings of this study are available in the paper and Supplementary Information. Source data are provided with this paper.

## Source data

Source Data

## Materials availability

Correspondence and requests for materials should be addressed to J.W
