# Supplementary material for: Selective photocatalytic CO2 reduction in aerobic environment by microporous Pd-porphyrin-based polymers coated hollow TiO2
Source: Nat Commun. 2022 Mar 17;13:1400. doi: 10.1038/s41467-022-29102-0 (PMC8930982; doi:10.1038/s41467-022-29102-0)
Supplement: Supplementary file 1 — Supplementary Information [file 41467_2022_29102_MOESM1_ESM.pdf]

Supplementary Information

Selective photocatalytic CO_2 reduction in aerobic environment by microporous Pd-porphyrin-based polymers coated hollow TiO_2

Ma et al.

Table of Contents

Supplementary Fig. 1 Long-term test of photocatalytic CO_2 reduction over photocatalysts. S5

Supplementary Fig. 2 Photocatalytic CO_2 reduction in different gas environment. S5

Supplementary Fig. 3 GC-MS of gas products after the photocatalytic reaction over Pd-HPP-TiO_2. S6

Supplementary Fig. 4 Electrochemical impedance spectra (EIS) of as-prepared photocatalysts. S7

Supplementary Fig. 5. Photoluminescence (PL) spectra of as-prepared photocatalysts. S8

Supplementary Fig. 6 UV–vis diffuse reflectance spectra of as-prepared photocatalysts. S9

Supplementary Fig. 7 The evolution rates of CH_4 and CO in pure CO_2 under visible light irradiation ($\lambda \geq 420$ nm). S9

Supplementary Fig. 8 Effect of O_2 existence on the photocatalytic CO_2 reduction. S10

Supplementary Fig. 9 Effect of CO_2 concentration on the photocatalytic CO_2 reduction. S10

Supplementary Fig. 10 CO_2 adsorption and desorption isotherms at 273 K. S11

Supplementary Fig. 11 CO_2 adsorption and desorption isotherms of porous Pd-HPP-TiO_2 composites at 273 K. S11

Supplementary Fig. 12 The evolution rates of CH_4 and CO over porous Pd-HPP-TiO_2 composites. S12

Supplementary Fig. 13 Comparisons in CO_2 and O_2 adsorption isotherms at 273 K. S12

Supplementary Fig. 14 SEM images. S13

Supplementary Fig. 15 Low-magnified TEM image of Pd-HPP-TiO_2. S13

Supplementary Fig. 16 FT-IR spectra of of as-prepared photocatalysts. S14

Supplementary Fig. 17 XPS spectra of hollow TiO_2, Pd/TiO_2, Pd-HPP, and Pd-HPP-TiO_2. S15

Supplementary Fig. 18 In situ DRIFTS tests in the region of 3500-3800 cm^{-1}. S16

Supplementary Fig. 19 Cycling test of photocatalytic CO_2 reduction. S16

Supplementary Fig.e 20 Thermogravimetric analysis of Pd-HPP at a heating rate of 10 °C min^{-1} in air. S17

Supplementary Fig. 21 Changes in the FT-IR spectra of Pd-HPP-TiO_2 by the photocatalysis. S17

Supplementary Fig. 22 XRD patterns of Pd-HPP-TiO_2 before and after the photocatalytic reaction. S18

Supplementary Fig. 23 XPS spectra of Pd-HPP-TiO_2 before and after the photocatalytic reaction. S19

Supplementary Fig. 24 Full spectrum of light source. S20

Supplementary Table 1 Comparison of the photocatalytic activity of Pd-HPP-TiO_2 with the recently reported catalysts for CO_2 reduction under similar reaction conditions S20

Supplementary Table 2 Comparisons of photocatalytic CO_2 conversion in air reported recently. S21

Supplementary Table 3 Conversion yield of CO_2 in 0.15 vol% CO_2/N_2 and air for Pd-HPP-TiO_2 and Pd/TiO_2. S21

Supplementary Table 4 The porosity parameters and gas uptake of as-prepared photocatalysts. S22

Supplementary Table 5 Comparison of the molar ratio of porphyrin/CO_2 in different photocatalysts. S22

Supplementary Table 6 EXAFS fitting parameters at the Pd K-edge (S_0^2= 0.829). S23

Supplementary Table 7 Pd and TiO_2 contents in Pd-HPP-TiO_2 and Pd/TiO_2 based on the ICP-MS measurements. S23

Supplementary References S23

Supplementary Figures

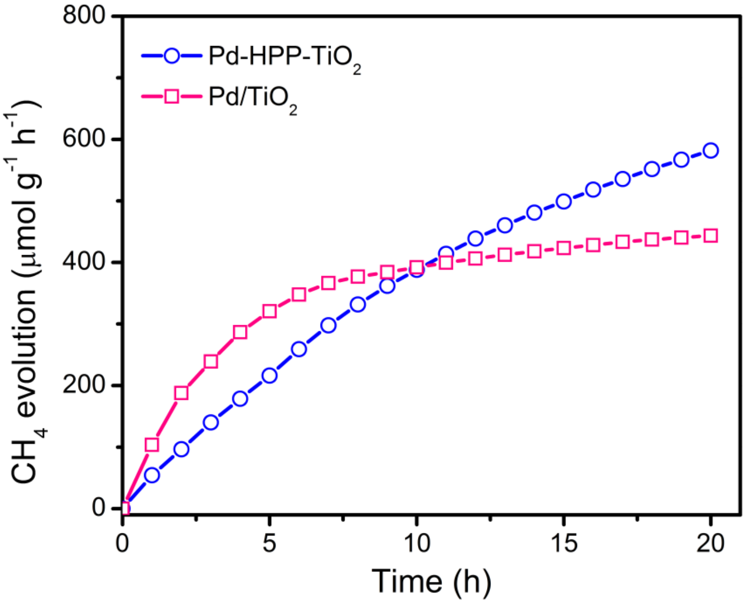

Supplementary Fig. 1 Long-term test of photocatalytic CO_2 reduction over photocatalysts. (a) Pd-HPP-TiO_2 and (b) Pd/TiO_2.

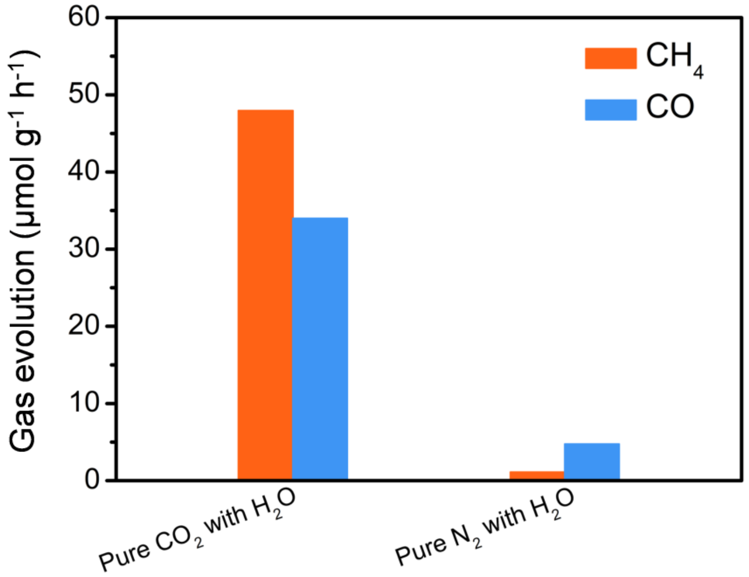

Supplementary Fig. 2 Photocatalytic CO_2 reduction in different gas environment. The evolution rates of CH_4 and CO over Pd-HPP-TiO_2 in pure CO_2 or N_2 with H_2O.

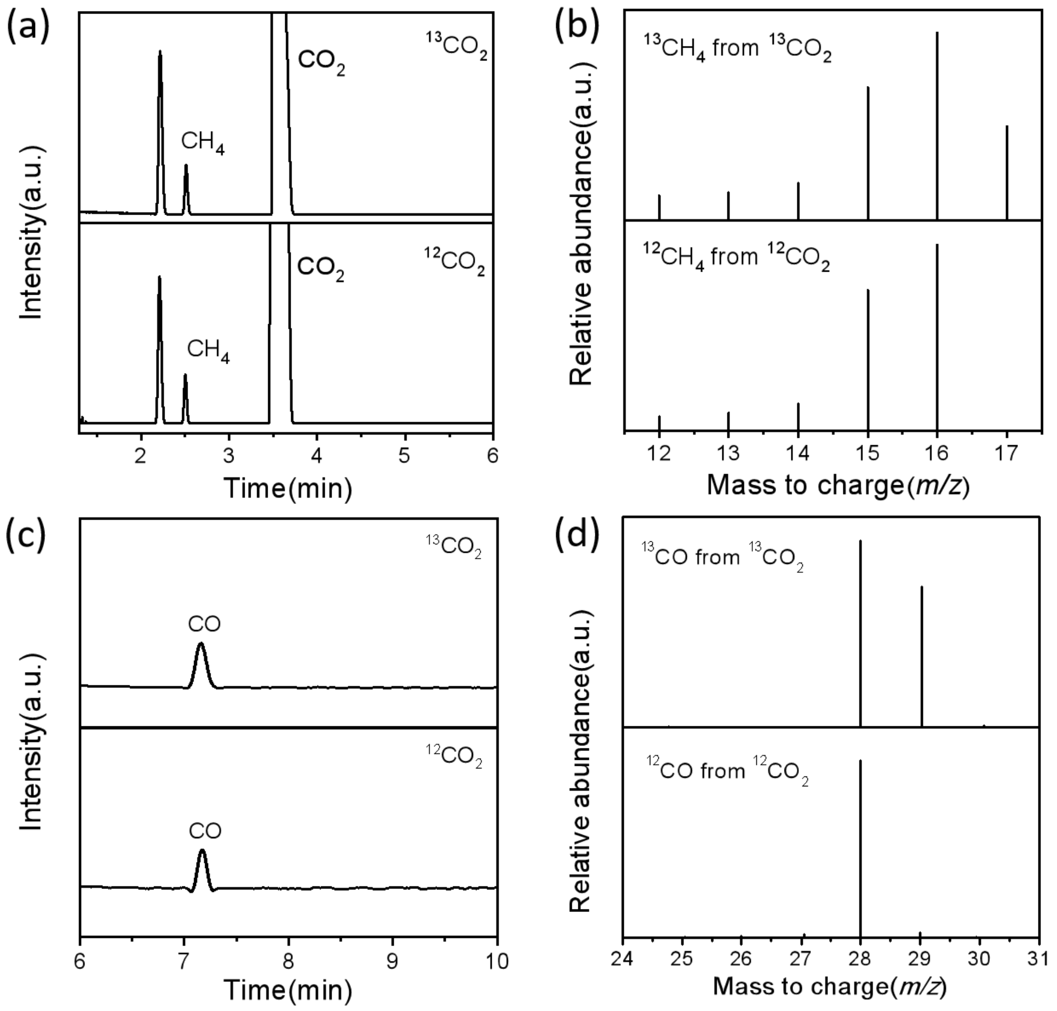

Supplementary Fig. 3 GC-MS of gas products after the photocatalytic reaction over Pd-HPP-TiO$_2$. (a, c) GC charts and (b, d) MS of CH_4 and CO produced from the isotopically labelled $^{13}CO_2$ (^{13}C enrichment of $\geq$ 97%) and $^{12}CO_2$ used as the reactant.

The appearance of m/z = 17 reveals that the produced $^{13}CH_4$ indeed originated from the $^{13}CO_2$ reduction over Pd-HPP-TiO$_2$. The peaks at m/z = 16 and 15 are assigned to the fragments of $^{13}CH_4$. The peaks at m/z = 29 reveals that the produced ^{13}CO originated from $^{13}CO_2$ reduction over Pd-HPP-TiO$_2$. The residual and pre-adsorbed $^{12}CO_2$ on Pd-HPP-TiO$_2$ and glass surface of the reactor from air are not negligible in contributing to m/z = 28, 16 and 15.

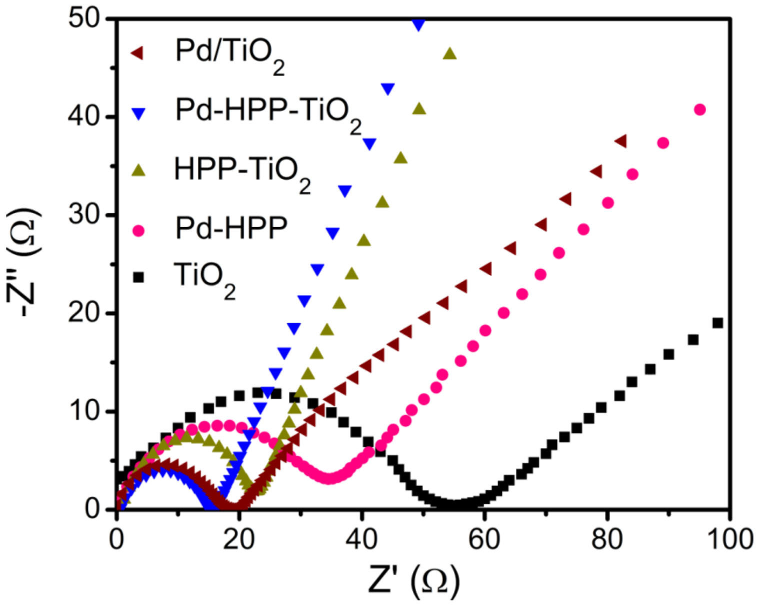

Supplementary Fig. 4 Electrochemical impedance spectra (EIS) of the as-prepared samples. Work electrode: indium-tin-oxide (ITO) glass dip-coated by the samples; reference electrode: Ag/AgCl electrode; counter electrode: platinum wire; electrolyte: 0.1 M KCl aqueous solution containing 5 mM Fe $(CN)_6^{3-}$/Fe $(CN)_6^{4-}$.

The size of the semicircle at high frequencies in the EIS Nyquist diagram represents the charge transfer resistance (R_{ct}). Pd-HPP and TiO_2 show large R_{ct} due to lower electronic conductivities than that of Pd-HP-TiO_2, suggesting the efficient electron transfer between TiO_2 and Pd-HPP with effective interfacial interaction and efficient charge transfer in Pd-HPP.

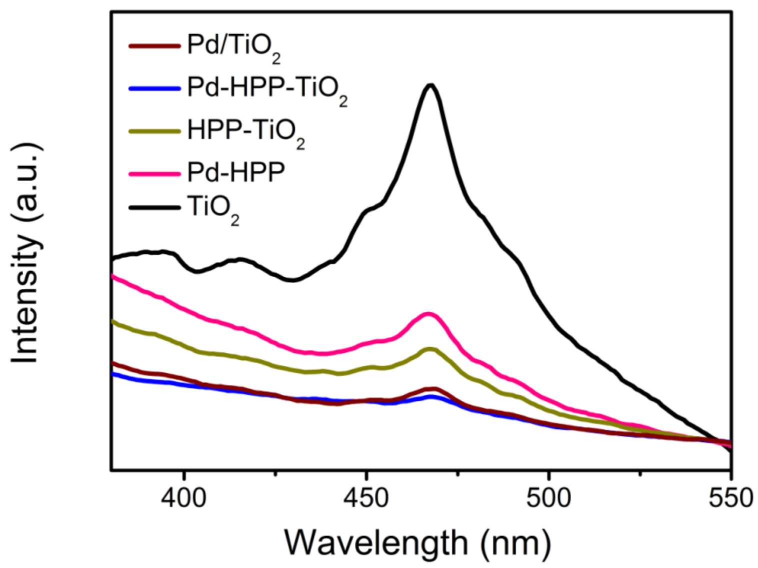

Supplementary Fig. 5 Photoluminescence (PL) spectra of as-prepared photocatalysts. Those of Pd/TiO_2, Pd-HPP-TiO_2, HPP-TiO_2, Pd-HPP, and TiO_2 at 360 nm excitation were compared.

Hollow TiO_2 exhibited a strong and broad emission band in the region of 380-550 nm, assigned tothe surface defects[1]. The largest PL quenching was observed for Pd-HPP-TiO_2, indicating a decrease of charge recombination because of rapid electron transfer from TiO_2 to Pd-HPP.

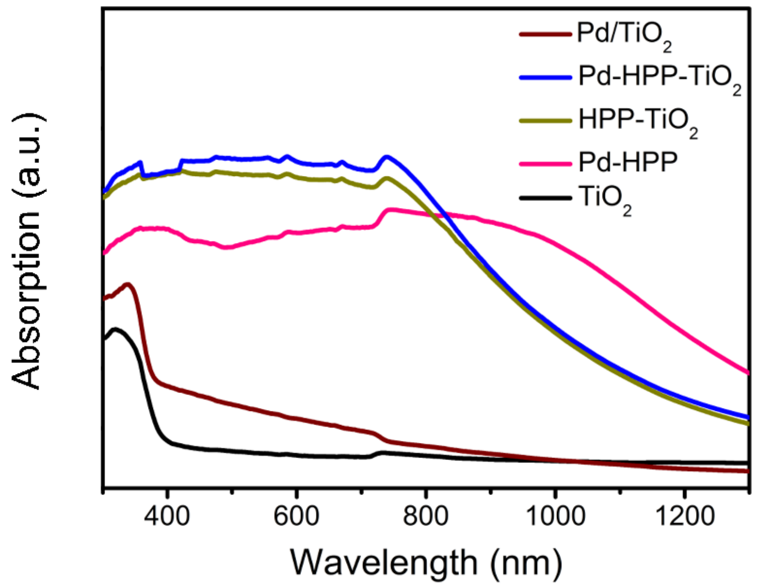

Supplementary Fig. 6 UV–vis diffuse reflectance spectra of as-prepared photocatalysts. Those of Pd/TiO_2, Pd-HPP-TiO_2, HPP-TiO_2, Pd-HPP, and TiO_2 were compared.

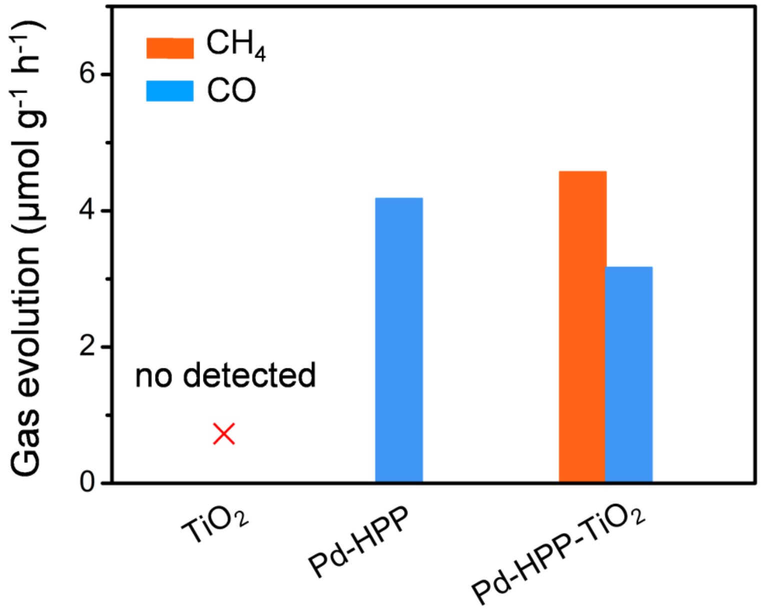

Supplementary Fig. 7 The evolution rates of CH_4 and CO in pure CO_2 under visible light irradiation ($\lambda \geq 420$ nm). The rates over TiO_2, Pd-HPP, and Pd-HPP-TiO_2 were compared.

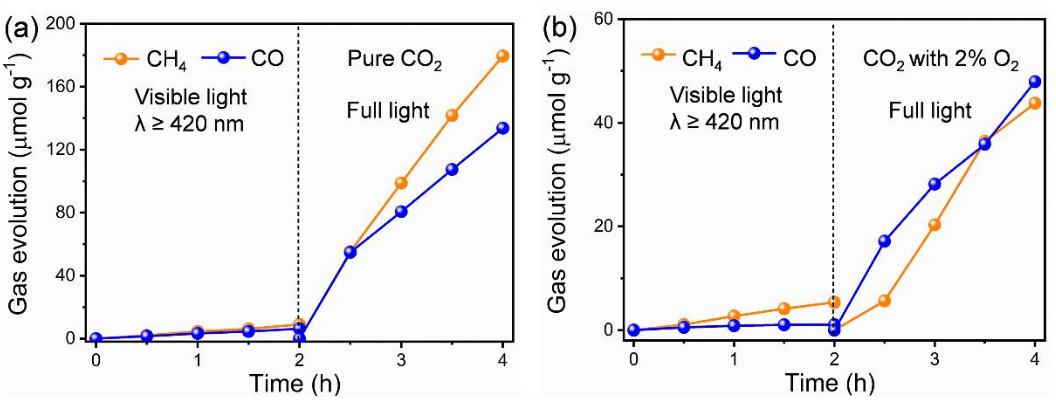

Supplementary Fig. 8 Effect of O_2 existence on the photocatalytic CO_2 reduction. The evolution rates of CH_4 and CO over Pd-HPP-TiO_2 photocatalyst under visible light irradiation ($\lambda \geq$ 420 nm) and full light irradiation. (a) in pure CO_2 (b) in CO_2 with 2 vol% O_2.

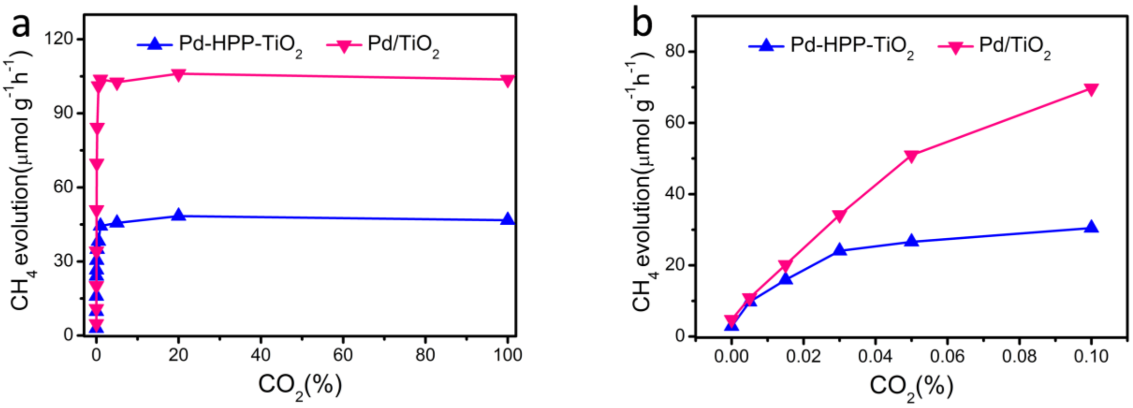

Supplementary Fig. 9 Effect of CO_2 concentration on the photocatalytic CO_2 reduction. The CH_4 evolution rate in CO_2/N_2 synthetic gas over Pd/TiO_2 and Pd-HPP-TiO_2 photocatalysts.

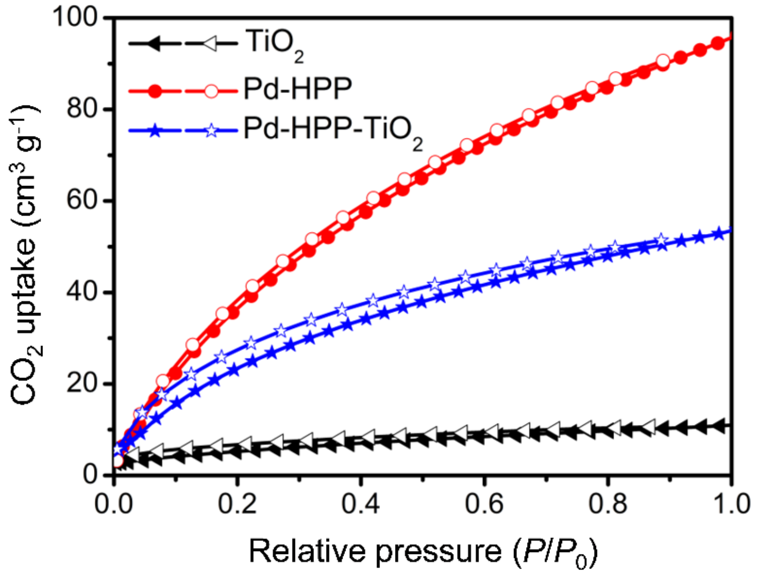

Supplementary Fig. 10 CO_2 adsorption and desorption isotherms at 273 K. Those of hollow TiO_2, Pd-HPP, and Pd-HPP-TiO_2 were compared.

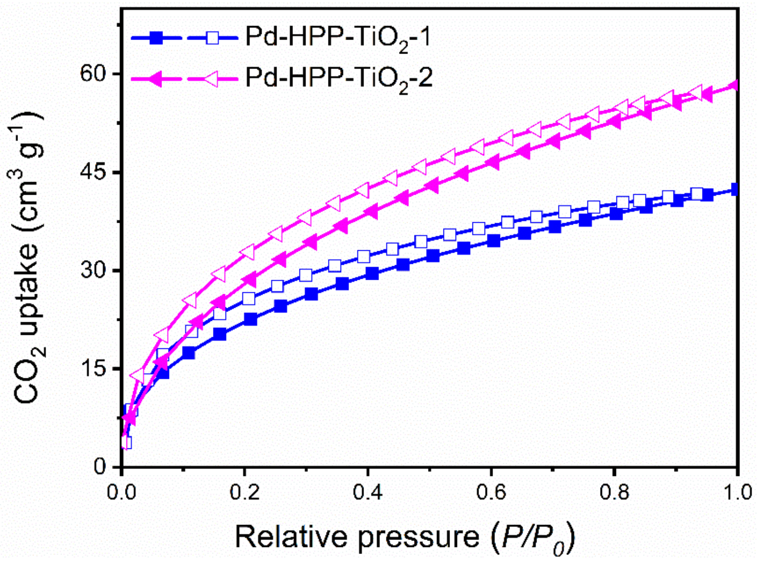

Supplementary Fig. 11 CO_2 adsorption and desorption isotherms of porous Pd-HPP-TiO_2 composites at 273 K. Pd-HPP-TiO_2-1 was synthesized by adding 15 mg of TPP monomer. Pd-HPP-TiO_2-2 was synthesized by adding 45 mg of TPP monomer.

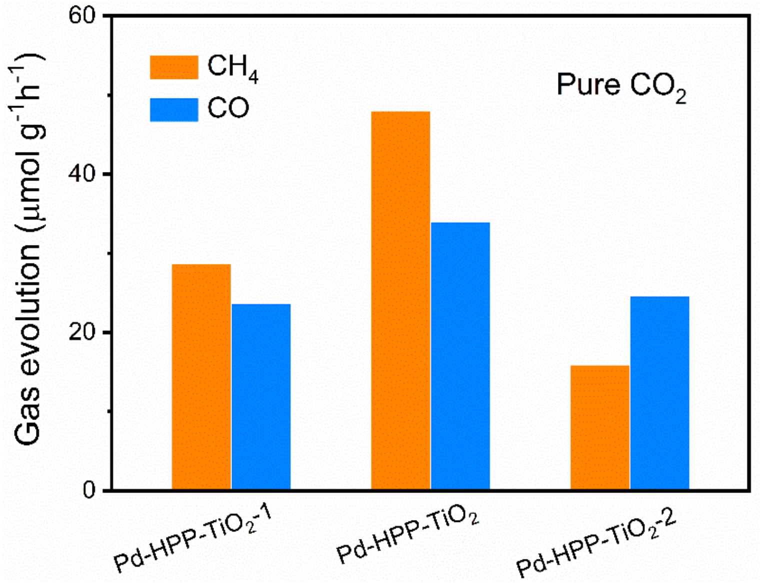

Supplementary Fig. 12 The evolution rates of CH_4 and CO over porous Pd-HPP-TiO_2 composites. Pd-HPP-TiO_2-1 was synthesized by adding 15 mg of TPP monomer. Pd-HPP-TiO_2 was synthesized by adding 30 mg of TPP monomer. Pd-HPP-TiO_2-2 was synthesized by adding 45 mg of TPP monomer.

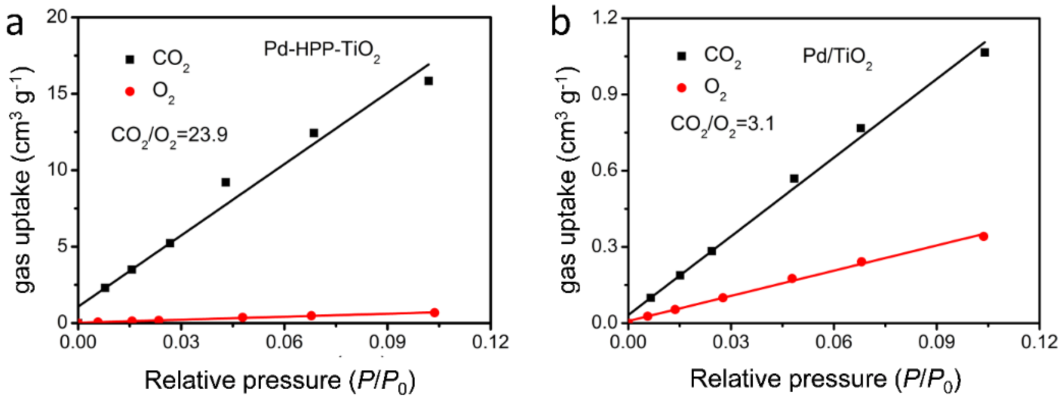

Supplementary Fig. 13 Comparisons in CO_2 and O_2 adsorption isotherms at 273 K. (a) Pd-HPP-TiO_2 and (b) Pd/TiO_2. The CO_2/O_2 selectivity ratio was calculated by the initial slopes of adsorption isotherms in the pressure less than 0.1[2,3].

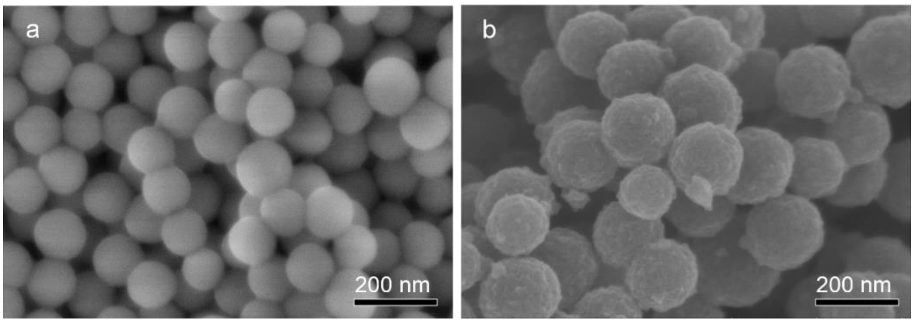

Supplementary Fig. 14 SEM images. **(a)** SiO_2 spheres and **(b)** core-shell SiO_2@TiO_2.

The SEM image indicates that SiO_2 spheres were covered by TiO_2 to formcore-shell SiO_2@TiO_2.

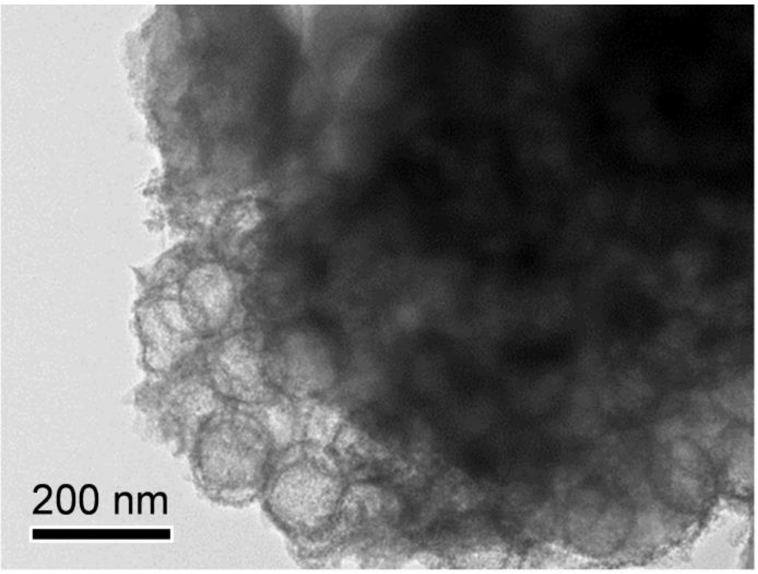

Supplementary Fig. 15 Low-magnified TEM image of Pd-HPP-TiO_2.

After step I and II in Figure 1, hollow TiO_2 were uniformly covered by Pd-HPP as shown in transmission electron microscopy (TEM) images (Figures 4b and S15).

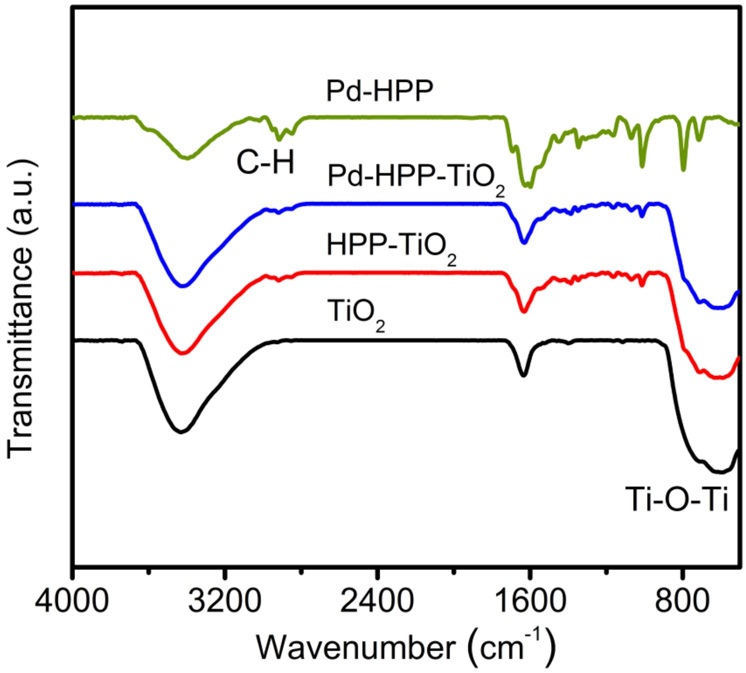

Supplementary Fig. 16 FT-IR spectra of of as-prepared photocatalysts. Those of hollow TiO_2, HPP-TiO_2, Pd-HPP, and Pd-HPP-TiO_2 were compared.

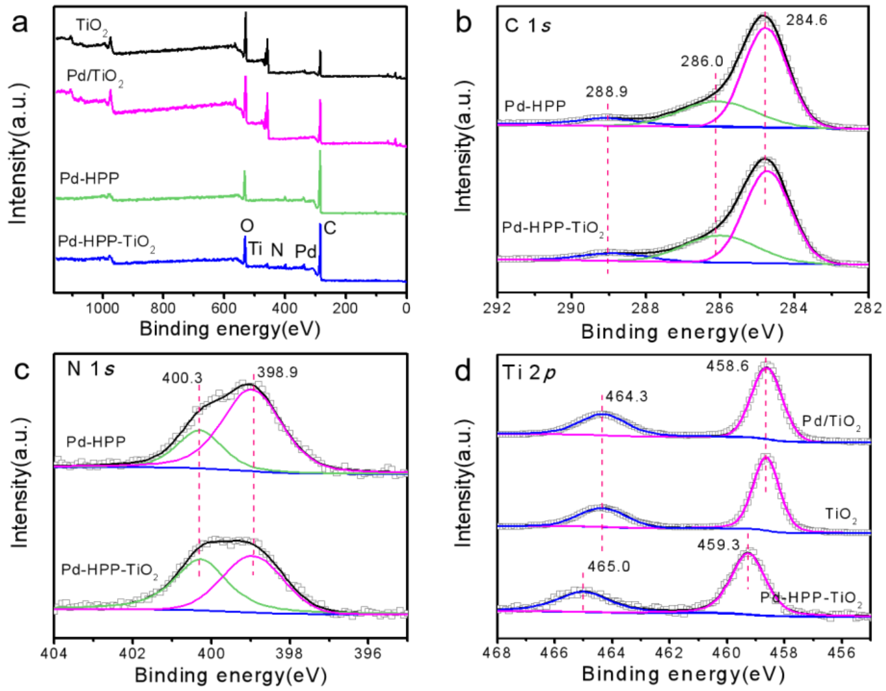

Supplementary Fig. 17 XPS spectra of hollow TiO_2, Pd/TiO_2, Pd-HPP, and Pd-HPP-TiO_2. **(a)** XPS survey spectra. High-resolution XPS spectra of **(b)** C 1*s*, **(c)** N 1*s*, and **(d)** Ti 2*p* in the corresponding samples.

The high-resolution C 1*s* spectra were deconvoluted to three peaks at 288.9, 286.0, and 284.6 eV, assigned to Cs of C-N, C=N, and C-C of HPP, respectively. The N 1*s* spectra show two peaks at 400.3 and 398.9 eV, corresponding to pyrrolic-N and Pd-N in Pd-HPP. Combined with the presence of Pd(II) in Pd 3*d* spectra (Figure 5b), it can be deduced that Pd(II) coordinates with the porphyrin. The intensity of the Pd-N peak decreased in Pd-HPP-TiO_2, presumably due to the interaction between TiO_2 and N of the porphyrin. In the high-resolution Ti 2*p* spectra, two peaks were observed at 464.3 and 458.6 eV, corresponding to Ti $2p_{3/2}$ and Ti $2p_{1/2}$. The interaction between Pd-HPP and TiO_2 causes higher-energy shifts of ~0.7 eV for two peaks.

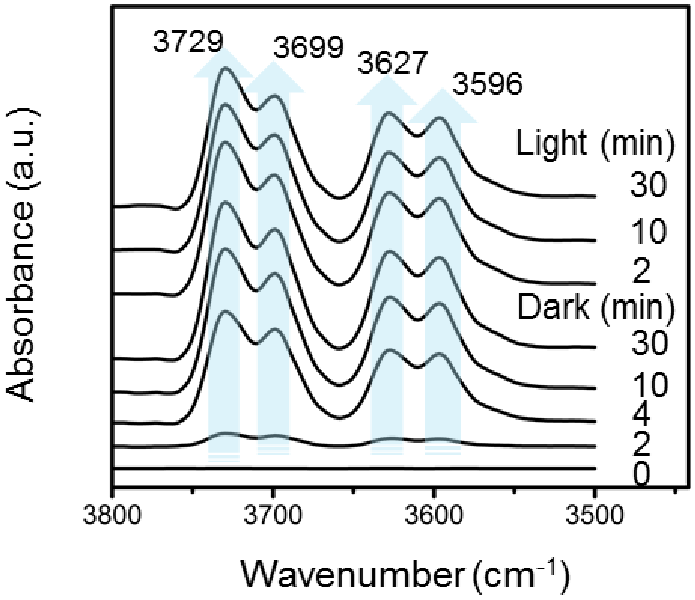

Supplementary Fig. 18 In situ DRIFTS tests in the region of 3500-3800 cm^{-1}. Gas adsorption and photocatalytic reaction over Pd-HPP-TiO_2 in the dark and under UV-visible light irradiation.

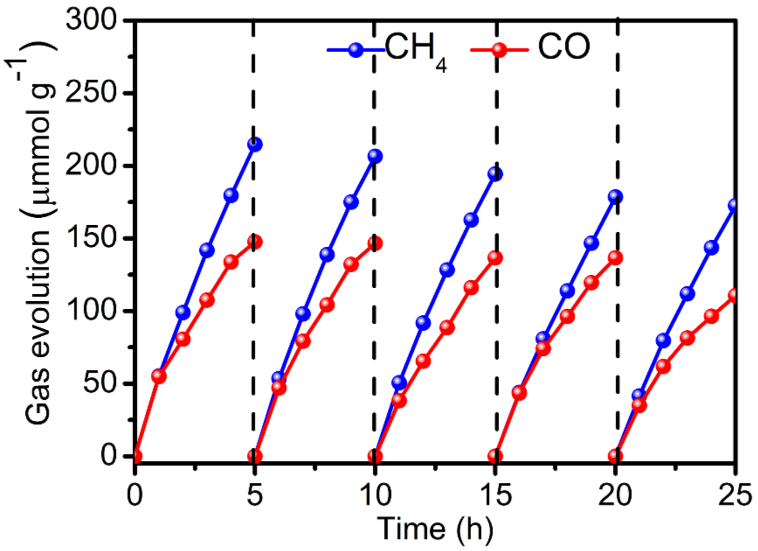

Supplementary Fig. 19 Cycling test of photocatalytic CO_2 reduction. The CH_4 and CO evolution over Pd-HPP-TiO_2 within 5 cycles.

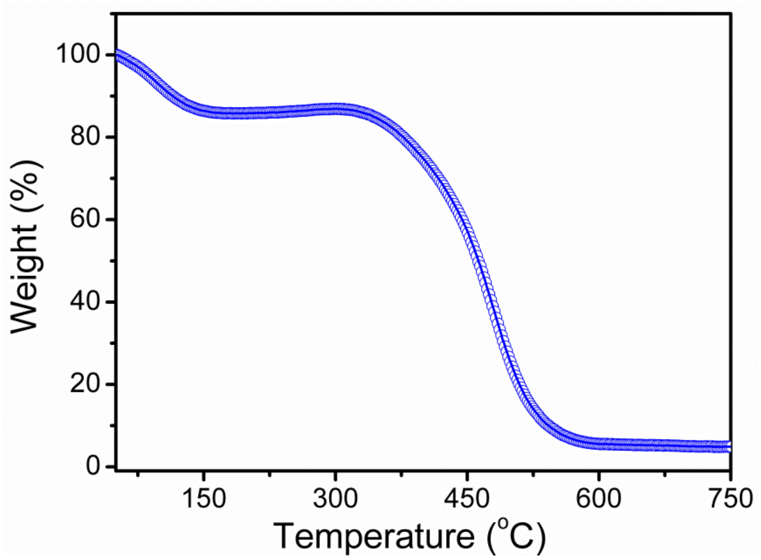

Supplementary Fig. 20 Thermogravimetric analysis of Pd-HPP at a heating rate of 10 ℃ min^{-1} in air.

The thermostability of Pd-HPP was investigated by thermogravimetric analysis. Upon heating, the weight loss of about 5.1 % below 150 ℃ came from the adsorbed water and other gaseous molecules. The decomposition of HPP was observed when Pd-HPP was heated at higher than 330 ℃.

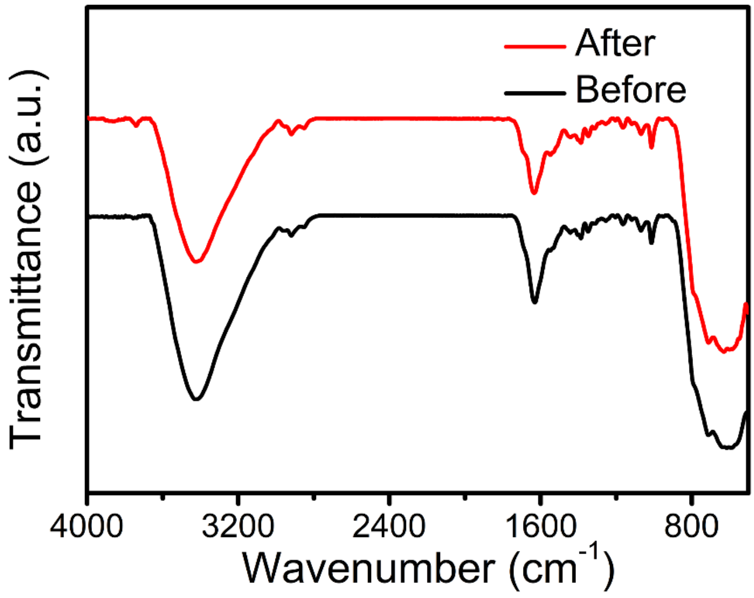

Supplementary Fig. 21 Changes in the FT-IR spectra of Pd-HPP-TiO_2 by the photocatalysis. Comparison between before and after the photocatalytic reaction.

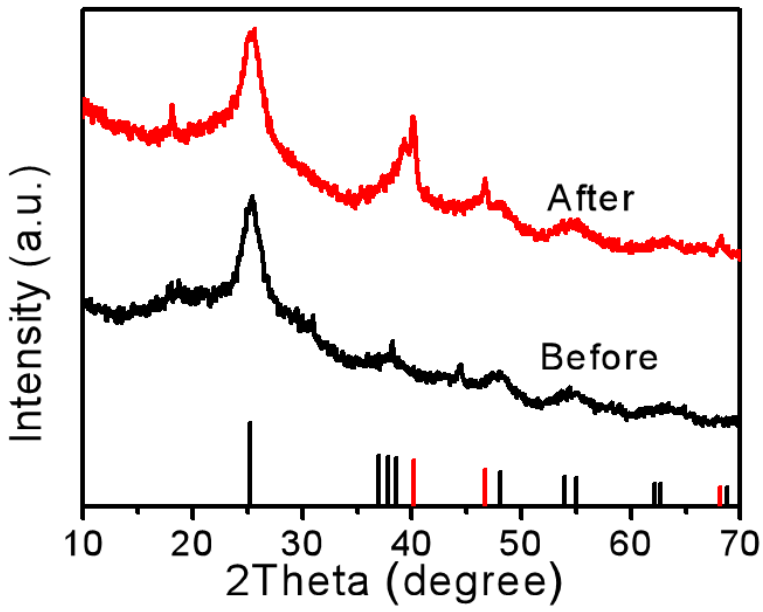

Supplementary Fig. 22 XRD patterns of Pd-HPP-TiO_2 before and after the photocatalytic reaction.

The vertical lines are the position and intensity of anatase TiO_2 (black line, JCPDS 21-1272) and Pd (red line, JCPDS 46-1043).

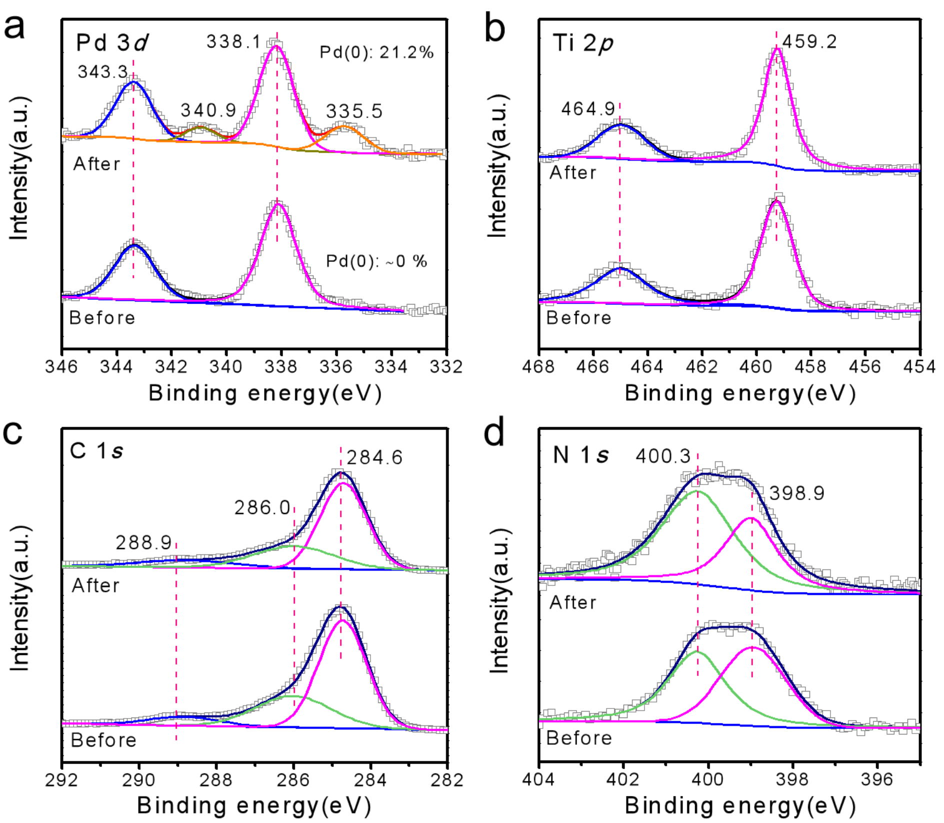

Supplementary Fig. 23 XPS spectra of Pd-HPP-TiO_2 before and after the photocatalytic reaction. (a) Pd 3*d*, (b) Ti 2*p* , (c) C 1*s*, and (d) N 1*s*. The ratio of Pd (II) and Pd (0) was calculated for Pd-HPP-TiO_2 before and after the reaction.

After the photocatalytic reaction, Pd existed as Pd(II) in ~78.8%, i.e. 21.2% of Pd(II) of Pd-HPP-TiO_2 changed to Pd(0). The largely remained Pd(II) in the composite can be ascribed to the confinement effect and coordination with porphyrin of HPP. No significant difference was observed in the spectra of Ti 2*p*, C1*s*, and N1*s*, indicating the durability of HPP and TiO_2.

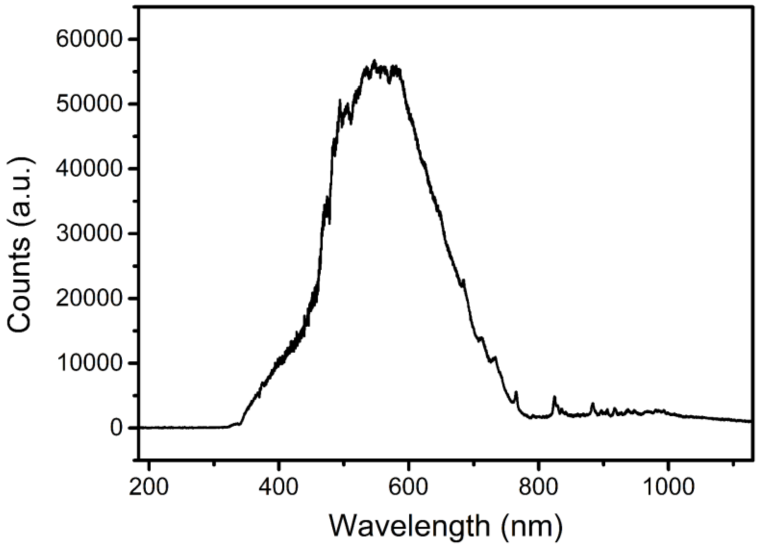

Supplementary Fig. 24 Full spectrum of light source. Light: Xe lamp (PLS-SXE300D, Beijing Perfectlight, China).

Supplementary Table 1 Comparison of the photocatalytic activity of Pd-HPP-TiO_2 with the recently reported catalysts for CO_2 reduction under similar reaction conditions (gas-solid reaction just using H_2O as electron donor).

Photocatalysts	Products (μmol g^{-1} h^{-1})		Ref
	CH_4	CO	
Pyrazolyl Porphyrinic Ni-MOF	10.1	6.0	5
1%B/g-C_3N_4	0.16	-	6
a-Fe_2O_3/Amine-RGO/$CsPbBr_3$	28.5	6.0	7
V_o-$BiOIO_3$	-	16.33	8
N-Doped Graphene on CdS	0.33	2.59	9
V_s-$CuIn_5S_8$	8.7	-	10
Porous CoO@N-GCs	10.03	5.16	11
Hypercrosslinked Polymers-3	0.30	5.10	12
Porphyrin Based COF	-	24.6	13
Triphenylamine Based CMPs	-	37.15	14
ZnSe/CdS DORs	-	11.3	15
Pd-HPP-TiO_2	48	34	This work

Supplementary Table 2 Comparisons of photocatalytic CO_2 conversion in air reported recently.

Photocatalysts	S_{BET} (m^2 g^{-1})	Irradiation light, additives	Products (μmol g^{-1} h^{-1})	Conversion yield of CO_2	References
$Rb_{0.33}WO_3$	5.0	UV-vis, H_2O	CH_3OH: 3.73; CO: 0.07; CH_4: 0.02; HCHO: 1.05	4.3 % (4 h)	16
Mo-doped $Cs_{0.33}WO_3$	--	UV-vis, H_2O,	CO: 3.4; CH_3OH: 1	--	17
Ionic liquid([P_{4444}][p-2-O])-pyrene-based polymer	23.9	λ > 420 nm, TEOA	CO: 47.37	--	18
Pd/TiO_2 (control)	61	UV-vis, H_2O	CH_4: 2.7; CO: --	2.6 % (2 h)	This work
Pd-HPP-TiO_2	323.0	UV-vis, H_2O	CH_4: 12.2; CO: 4.9	12 % (2 h)	This work

Supplementary Table 3 Conversion yield of CO_2 in 0.15 vol% CO_2/N_2 and air for Pd-HPP-TiO_2 and Pd/TiO_2.

Photocatalsts	Atmosphere	CO_2 in the reactor (ppm) (CO_2 conversion rate (%))		
		Before reaction	Irradiation for 1 h	Irradiation for 2 h
Pd/TiO_2	0.15 vol% CO_2/N_2	1384 (-)	1297 (6.3)	1224 (12)
	Air	332 (-)	326 (1.9)	323 (2.7)
Pd-HPP-TiO_2	0.15 vol% CO_2/N_2	1362 (-)	1322 (3.0)	1285 (5.7)
	Air	319 (-)	297 (7.2)	282 (12)

Supplementary Table 4 The porosity parameters and gas uptake of as-prepared photocatalysts.

Photocatalysts	S_{BET}[a] (m^2 g^{-1})	Pore volume[b] (cm^3 g^{-1})	Micropore volume[c] ($cm^3 g^{-1}$)	CO_2 uptake[d] (cm^3 g^{-1})	O_2 uptake[e] (cm^3 g^{-1})
Pd-HPP	1252	0.71	0.61	96	-
TiO_2	75	0.13	-	11	-
Pd-HPP-TiO_2	323	0.29	0.22	54	4.3
Pd/TiO_2	61	0.1	-	6.5	2.8

[a] Calculated from the N_2 adsorption-desorption isotherms at 77.3 K. [b]Calculated from N_2 isotherm at P/P_0=0.995 and 77.3 K. [c]Calculated from the N_2isotherm at P/P_0=0.050. [d]Calculated from the CO_2 adsorption-desorption isotherms at 1.00 bar and 273 K. [e]Calculated from the O_2 adsorption-desorption isotherms at 1.00 bar and 273 K.

Supplementary Table 5 Comparison of the molar ratio of porphyrin/CO_2 in different photocatalysts.

Photocatalysts	Pd-HPP Content (wt%)	Porphyrin Content (wt%)	CO_2 uptake (cm^3 g^{-1})[d]	Porphyrin/CO_2 (molar ratio)
Pd-HPP	100	70.5	96	1:3.7
Pd-HPP-TiO_2-1[a]	53.8	37.9	42.4	1:3.1
Pd-HPP-TiO_2[b]	65.6	46.2	54	1:3.2
Pd-HPP-TiO_2-2[c]	74.9	52.8	58.3	1:3.0

[a] Pd-HPP-TiO_2-1 was synthesized by adding 15 mg of TPP monomer.

[b] Pd-HPP-TiO_2 was synthesized by adding 30 mg of TPP monomer.

[c] Pd-HPP-TiO_2-2 was synthesized by adding 45 mg of TPP monomer.

[d]Calculated from the CO_2 adsorption-desorption isotherms at 1.00 bar and 273 K.

Supplementary Table 6 EXAFS fitting parameters at the Pd K-edge (S_0^2= 0.829)[a].

Photocatalyst	Shell	*N*	*R*(Å)	σ^2 (Å^2)	ΔE_0 (eV)	*R* factor[b]
Pd-HPP-TiO_2	Pd-N	3.9	2.03	0.0043	2.6	0.0001

[a]S_0^2 was set to 0.829, according to the experimental EXAFS fit of Pd foil reference by fixing CN as the known crystallographic value.

[b]*N*: coordination numbers; *R*: bond distance; σ^2: Debye-Waller factor; ΔE_0: the inner potential correction. *R* factor: goodness of fit.

Supplementary Table 7 Pd and TiO_2 contents in Pd-HPP-TiO_2 and Pd/TiO_2 based on the ICP-MS measurements.

Photocatalysts	Pd content (wt%)	TiO_2 content (wt%)
Pd-HPP-TiO_2	2.72	34.4
Pd/TiO_2	2.68	97.3

Supplementary References

1. Ma, Y. *et al.* Assembling ultrafine TiO_2 nanoparticles on UiO-66 octahedrons to promote selective photocatalytic conversion of CO_2 to CH_4 at a low concentration. *Appl. Catal. B-Environ.* **270**, 118856 (2020).
2. Wang, S. *et al.* Porous hypercrosslinked polymer-TiO_2-graphene composite photocatalysts for visible-light-driven CO_2 conversion. *Nat. Commun.* **10**, 626 (2019).
3. Saleh, M., Lee, H. M., Kemp, K. C., Kim, K. S. Highly stable CO_2/N_2 and CO_2/CH_4 selectivity in hyper-cross-linked heterocyclic porous polymers. *ACS Appl. Mater. Interfaces* **6**, 7325−7333 (2014).
4. Lu, C. *et al.* Atomic Ni anchored covalent triazine framework as high efficient electrocatalyst for carbon dioxide conversion. *Adv. Funct. Mater.* **29**, 1806884 (2019).
5. Fang, Z. *et al.* Boosting interfacial charge-transfer kinetics for efficient overall CO_2 photoreduction via rational design of coordination spheres on metal−organic frameworks *J. Am. Chem. Soc.* **142**, 12515-12523 (2020).
6. Fu, J. *et al.* Graphitic carbon nitride with dopant induced charge localization for enhanced photoreduction of CO_2 to CH_4. *Adv. Sci.* **6**, 1900796 (2019).
7. Jiang, Y. *et al.* All-solid-state Z-scheme α-Fe_2O_3/amine-RGO/$CsPbBr_3$ hybrids for visible light-driven photocatalytic CO_2 reduction. *Chem* **6**, 766-780 (2020).
8. Chen, F. *et al.* Macroscopic spontaneous polarization and surface oxygen vacancies collaboratively boosting CO_2 photoreduction on $BiOIO_3$ single crystals. *Adv. Mater.* **32**, 1908350 (2020).

9. Bie, C. *et al.* In situ grown monolayer N-doped graphene on CdS hollow spheres with seamless contact for photocatalytic CO_2 reduction. *Adv. Mater.* **31**, 1902868 (2019).
10. Li, X. *et al*. Selective visible-light-driven photocatalytic CO_2 reduction to CH_4 mediated by atomically thin $CuIn_5S_8$ layers. *Nat. Energy* **4**, 690-699 (2019).
11. He, L., Zhang, W., Liu, S. & Zhao, Y. Three-dimensional porous N-doped graphitic carbon framework with embedded CoO for photocatalytic CO_2 reduction. *Appl. Catal. B: Environ.* **298**, 120546 (2021).
12. Schukraft, G. E. M. *et al.* Hypercrosslinked polymers as a photocatalytic platform for visible-light-driven CO_2 photoreduction using H_2O. *ChemSusChem* **14**, 1720-1727 (2021).
13. Wang, L. *et al*. Improved photoreduction of CO_2 with water by tuning the valence band of covalent organic frameworks. *ChemSusChem* **13**, 2973-2980 (2020).
14. Dai, C. *et al*. Triphenylamine based conjugated microporous polymers for selective photoreduction of CO_2 to CO under visible light. *Green Chem.* **21**, 6606-6610 (2019).
15. Xin, Z. *et al*. Rational design of dot-on-rod nanoheterostructure for photocatalytic CO_2 reduction: pivotal role of hole transfer and utilization. *Adv. Mater.* DOI: https://doi.org/10.1002/adma.202106662.
16 Wu, X. *et al.* Photocatalytic CO_2 conversion of $M_{0.33}WO_3$ directly from the air with high selectivity: insight into full spectrum-induced reaction mechanism. *J. Am. Chem. Soc.* **141**, 5267−5274 (2019).
17. Yi, L. *et al.* Tungsten bronze $Cs_{0.33}WO_3$ nanorods modified by molybdenum for improved photocatalytic CO_2 reduction directly from air. *Sci. Chin. Mater.* **63**, 2206–2214 (2020).
18. Chen, Y. *et al.* Visible-light-driven conversion of CO_2 from air to CO using an ionic liquid and a conjugated polymer. *Green Chem.* **19**, 5777−5781 (2017).
